# A premature ovarian insufficiency-associated LMD-3 point mutation impairs protein stability and causes autophagic-lysosomal dysfunction in *Caenorhabditis elegans*

**DOI:** 10.1371/journal.pone.0361215

**Published:** 2026-10-27

**Authors:** Yile Zhai, Tiantian Wang, Meixi Gong, Yuhan Liang, Wenfei Li, Xin Wang, Zhe Zhang

**Affiliations:** 1 Institute of Medical Sciences, The Second Qilu Hospital of Shandong University, Jinan, Shandong, China; 2 Department of Histology and Embryology, School of Basic Medical Sciences, Cheeloo College of Medicine, Shandong University, Jinan, Shandong, China; 3 State Key Laboratory for Conservation and Utilization of Bio-Resources in Yunnan, Yunnan University, Kunming, Yunnan, China; Al-Azhar University Faculty of Science for Boys in Cairo, EGYPT

## Abstract

Reproductive longevity decline characterizes both natural aging and premature ovarian insufficiency (POI). While our previous work established *C. elegans* LMD-3 as a critical regulator of reproductive capacity, the in vivo consequences of specific clinical mutations remain unclear. Here, we modeled the POI-associated human NCOA7 W804C mutation by engineering the orthologous W690C substitution into the *C. elegans lmd-3* locus. We demonstrate that this single-nucleotide substitution severely compromises LMD-3 protein stability and reduces transcript levels. This mutational destabilization is associated with profound systemic autophagic-lysosomal dysfunction, which impairs macromolecular degradation and leads to widespread proteotoxic accumulation of misfolded proteins. Coincident with these organismal defects, mutant animals exhibit an early-onset collapse in reproductive capacity driven by elevated germline apoptosis. Crucially, this pathology is consistent with reduced vitamin B12 availability; mecobalamin supplementation can mitigate the lysosomal block, restoring autophagic flux, proteostasis, and fecundity. These findings elucidate how a clinical mutation drives systemic lysosomal failure and reproductive decline, suggesting vitamin B12 supplementation as a promising metabolic intervention worth further translational exploration.

## Introduction

Reproductive senescence represents a hallmark of physiological aging, frequently manifesting as the earliest decline in female fecundity across diverse species, from *C. elegans* to humans [1,2]. In women, ovarian function diminishes prematurely compared to other somatic systems [3,4]. The acceleration of this process underlies premature ovarian insufficiency (POI), an increasingly global health concern [1,5]. A growing body of evidence connects this fertility loss to a breakdown in proteostasis, the integrated network safeguarding protein quality control [6]. Although cellular stress pathways and quality control mechanisms are conserved throughout evolution [7–9], the precise molecular triggers linking these systems to reproductive failure remain largely unmapped.

The nematode *C. elegans* offers an evolutionary conserved platform to dissect the molecular determinants of reproductive health [10,11]. Because its mid-life reproductive decline and oocyte quality deterioration mirror the human experience, it is highly suitable for investigating reproductive aging [2,12]. This decline is primarily orchestrated by the loss of germline integrity, a state contingent upon robust proteostasis, which is governed by specialized systems such as the lysosomal and autophagic machinery [13–15].

A key component of this protective network is the oxidation resistance 1 (OXR1) protein family, which includes OXR1 and its mammalian ortholog, nuclear receptor coactivator 7 (NCOA7) [16]. These evolutionarily conserved proteins are critical mediators of cellular stress responses [17,18]. Recently, NCOA7 has emerged as a regulator of ovarian longevity, with its orthologs exhibiting conserved protective effects against oxidative damage in zebrafish and *C. elegans* [19–21]. A defining feature of these proteins is the TLDc domain. This domain physically associates with V-type ATPase, the proton pump governing lysosomal acidification and proteolytic capacity [22,23]. These structural and functional parallels highlight the utility of *C. elegans* in decoding human reproductive pathology. We recently established that genetic ablation of *lmd-3*, the sole *C. elegans* ortholog of the NCOA7/OXR1 family, severely impairs reproductive capacity, yolk protein homeostasis, and lysosomal acidification [24].

However, while the phenotype resulting from the total loss of LMD-3 is well-defined, the biological consequences of clinical missense mutations, such as the POI-associated W804C mutation identified in the human NCOA7 TLDc domain [19], remain to be elucidated *in vivo*. It is currently unknown whether such site-specific amino acid substitutions function by destabilizing protein structure or by specifically disrupting essential protein-protein interactions.

In this study, we modeled the clinically relevant human mutation by engineering the orthologous W690C substitution into the *C. elegans lmd-3* locus to probe its biochemical and pathogenic consequences. Specifically, we sought to determine how this single-amino-acid substitution impacts LMD-3 protein stability, systemic autophagic-lysosomal function, and reproductive capacity. Furthermore, we investigated whether metabolic supplementation could mitigate these mutational defects, thereby providing new insights into the cellular mechanisms underlying early-onset reproductive decline.

## Results

### Genetic and structural evidence for a shared POI-associated W690C mutation in LMD-3

The *C. elegans* LMD-3 protein, which serves as the sole OXR1 family member in the nematode, possesses a domain architecture highly conserved with its human orthologs, OXR1 and NCOA7 (Fig 1A). This architecture is defined by three conserved LysM, GRAM, and TLDc domains. Despite the relatively low 26.46% overall amino acid sequence identity between LMD-3 and human NCOA7 [25], the functional domains and critical motifs are remarkably homologous (S1 Fig in S1 File).

**Fig 1:**
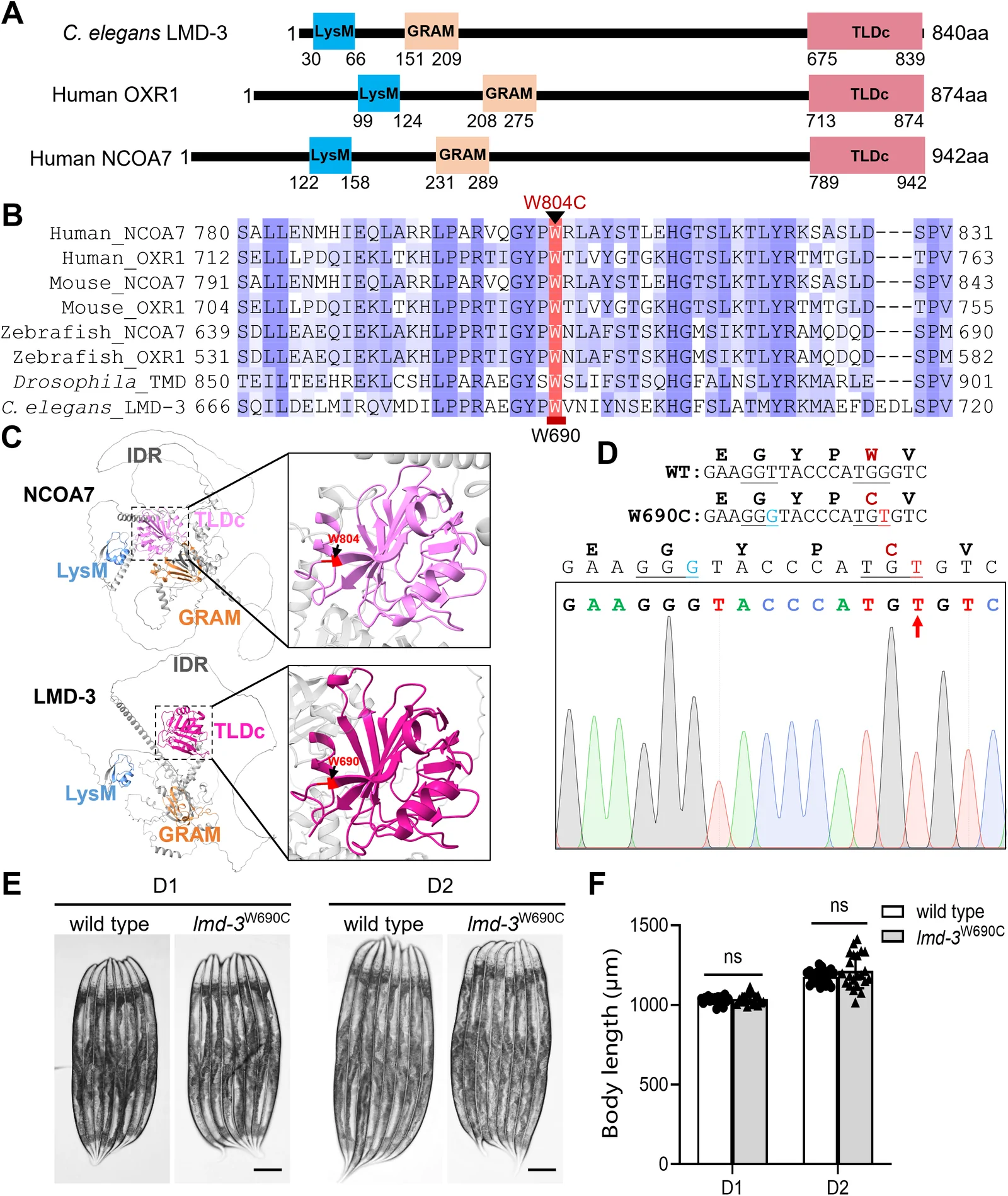
Characterization of the POI-associated W690C mutation in the conserved TLDc domain of *C. elegans* LMD-3. (A) Schematic representation of conserved domains in *C. elegans* LMD-3 and its human orthologs, OXR1 and NCOA7. The schematic diagrams illustrate the conserved LysM, GRAM, and TLDc domains. The total number of amino acids (aa) is indicated for each protein. (B) The conserved tryptophan residue (W804) associated with premature ovarian insufficiency (POI) is highly conserved across species. A multiple sequence alignment of NCOA7 orthologs from five different species demonstrates the conservation of POI-associated W804 residue in human NCOA7. The residue, corresponding to W690 in *C. elegans* LMD-3, is highlighted with a red arrowhead. (C) Comparative structural analysis of the TLDc domain from human NCOA7 and *C. elegans* LMD-3. The TLDc domains are shown in detail to highlight their conserved structure similarity. The intrinsically disordered region (IDR) is colored gray. The conserved POI-associated tryptophan residues (human W804 and *C. elegans* W690) are indicated by black arrows and are colored red in the detailed view. (D) Sequencing confirmation of the *lmd-3*^W690C^ knock-in allele. Representative DNA sequencing traces confirm the successful creation of the mutation. Red arrows indicate the target base change and the knock-in site. The resulting amino acid substitution (W to C) is highlighted in red, and the synonymous mutation introduced for screening is highlighted in light blue. (E, F) Development defects detection between wild-type and *lmd-3*^W690C^ animals at D1 and D2 adult stages with bright-field image (E) and quantification (F) of animal body lengths (n ≥ 17 for each group, unpaired t-tests: ns, no significant difference, with effect sizes of Cohen’s *d* = 0.199 for D1 and Cohen’s *d* = 0.480 for D2). Scale bar, 50 μm.

This conservation is further underscored by the POI-associated tryptophan residue (W804) located within the TLDc domain of human NCOA7 [19]. Sequence alignment revealed that this residue is strictly conserved across multiple species, corresponding to tryptophan 690 (W690) in *C. elegans* LMD-3 (Fig 1B and S1 Fig in S1 File) [26]. This remarkable conservation of a clinically relevant residue provides a strong genetic basis for a shared pathogenic mechanism, validating the *C. elegans* W690C mutant as a suitable model for investigating this specific POI-associated variant.

Following this genetic evidence, we sought to determine if this functional similarity was underpinned by structural conservation. Comparative structural analysis using AlphaFold3 revealed a striking similarity between the predicted *C. elegans* LMD-3 structure and its human NCOA7 ortholog (Fig 1C). Despite differences in unique α-helices and intrinsic disordered regions (IDRs), the core LysM, GRAM, and TLDc domains are highly conserved. This structural homology is particularly evident in the V-ATPase-interacting TLDc domain, which adopts an almost identical conformation and maintains the conserved W804/W690 site (Fig 1C and S1 Video). These results provide compelling structural insights that the TLDc-V-ATPase interaction is a central, conserved mechanism governing lysosomal activity and proteostasis across species.

Based on structural evaluations, we established an *in vivo* model of human POI by introducing the orthologous W690C mutation into the *C. elegans lmd-3* locus using CRISPR/Cas9 (Fig 1D and S1 Table). Preliminary phenotypic analysis of *lmd-3*^W690C^ homozygous mutants revealed no overt developmental defects, as confirmed by body length measurements comparable to those of wild-type animals (Fig 1E and 1F). The absence of significant developmental abnormalities, which is consistent with the localized reproductive pathology observed in human POI, confirms the *lmd-3*^W690C^ strain as a highly relevant *in vivo* model for dissecting the molecular mechanisms of this disease.

### The POI-associated W690C mutation impairs fecundity and germline integrity in *C. elegans*

The deleterious W804C mutation in human NCOA7 has been implicated in POI, primarily by enhancing oxidative stress-induced senescence in granulosa cells [19]. Building on the precise conservation of this tryptophan residue in *C. elegans* LMD-3 protein and our established findings regarding its role in antioxidant defense [20,27], we sought to determine if the orthologous *lmd-3*^*W690C*^ substitution would recapitulate the reproductive pathology observed in human patients. Our analysis revealed a significant reproductive deficit: the mutant strain exhibited an approximate 15% reduction in total progeny output compared to wild-type animals (Fig 2A, 2B; S2A and S2B Fig in S1 File). Recapitulating the early functional decline characteristic of POI, this reduction was particularly pronounced during the peak reproductive window (24–72 hours of adulthood).

**Fig 2:**
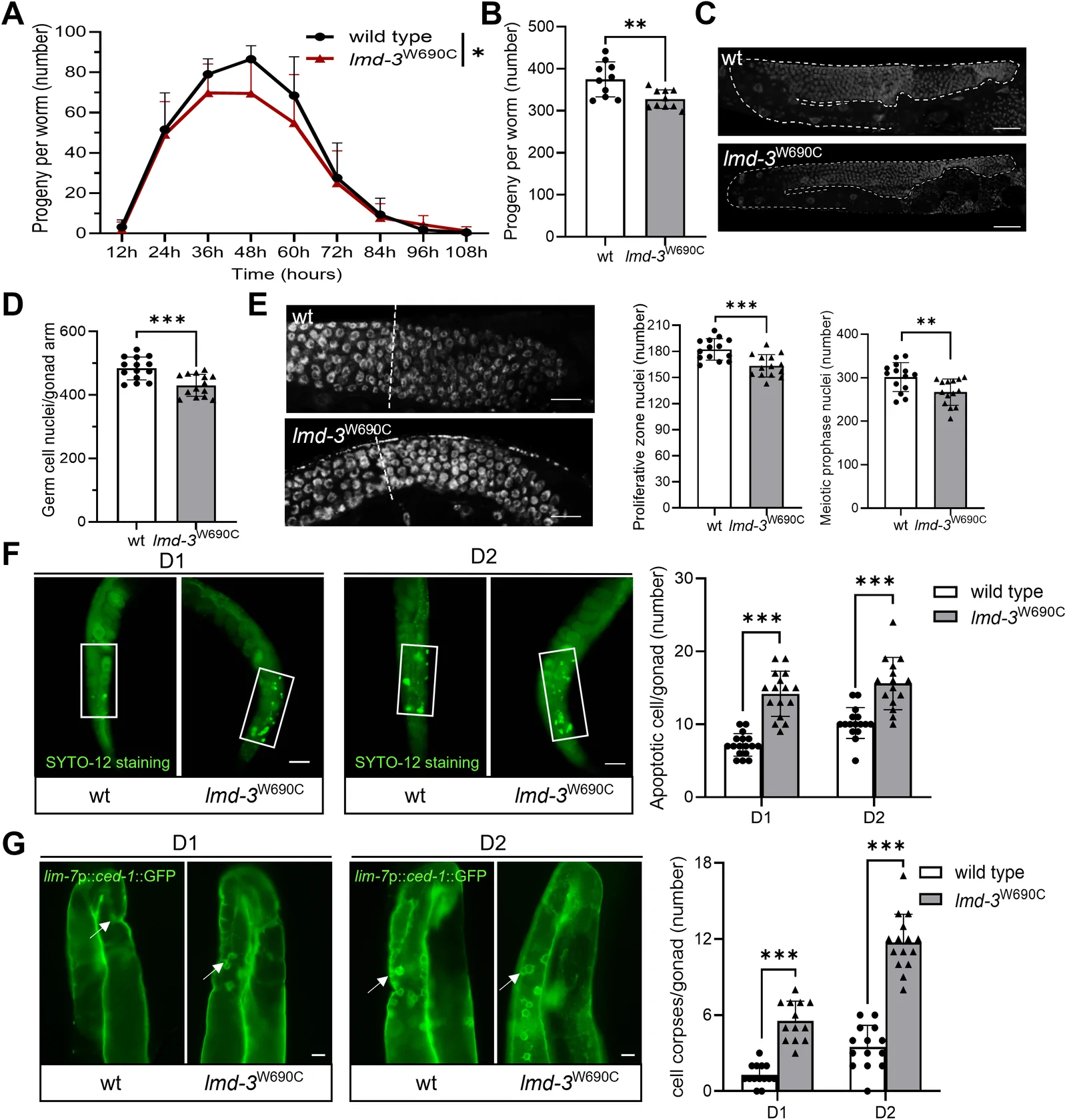
The POI-associated W690C mutation in *C. elegans* LMD-3 is associated with impaired fecundity and germline integrity. (A) Progeny production curves show the reproductive output of wild-type (black) and *lmd-3*^W690C^ (red) hermaphrodites (n ≥ 13 per group, *p < 0.05). (B) Total brood sizes indicate the overall reproductive ability of wild-type and *lmd-3*^W690C^ mutants. Data are presented as mean ± SEM (n = 10 per group, **p < 0.01, Cohen’s *d* = 1.414). (C, D) Representative fluorescence images (C) and quantification (D) of DAPI-stained total germline cells in wild-type and *lmd-3*^W690C^ mutants (n = 14 for each group, ***p < 0.001, Cohen’s *d* = 1.509). Scale bars, 50 μm. (E) Representative fluorescence images and quantification of DAPI staining for germline stem cells and meiotic cells in wild-type and *lmd-3*^W690C^ mutants (n = 14 for each group, **p < 0.01, ***p < 0.001, with effect sizes of Cohen’s *d* = 1.602 for proliferative zone nuclei and Cohen’s *d* = 0.994 for meiotic prophase nuclei). Scale bars, 20 μm. (F, G) Germline apoptosis in wild-type and *lmd-3*^W690C^ mutants with SYTO12 staining (F) and a *lim-7p::ced-1::GFP* reporter (G). White arrowheads indicate apoptotic cell corpses. n ≥ 13 per group; unpaired t-tests, ***p < 0.001. D1, Day 1 of adulthood; D2, Day 2 of adulthood, with effect sizes for (F) being Cohen’s *d* = 2.886 at D1 and Cohen’s *d* = 1.851 at D2, and for (G) being Cohen’s *d* = 3.445 at D1 and Cohen’s *d* = 4.147 at D2. Scale bars, 50 µm.

To dissect the cellular basis of this diminished brood size, we evaluated whether it originated from impaired embryo production or compromised embryonic viability. We quantified both egg-laying and hatching rates, revealing that *lmd-3*^W690C^ mutants laid 14.94% fewer eggs than wild-type controls (S2C Fig in S1 File). Conversely, the hatching rate remained robust and statistically indistinguishable between the two strains (S2C Fig in S1 File). This precise delineation suggests that the fecundity defect is driven by impaired oocyte production or ovulation, rather than by post-fertilization embryonic lethality.

Given that standard reproductive aging in *C. elegans* is typically precipitated by sperm depletion rather than an intrinsic decline in oocyte quality [28,29], cross-fertilization assays with *fem-1(hc17)* females were performed to test this [30]. These assays confirmed that sperm functionality remains fully intact in the mutants (S2D Fig in S1 File). Furthermore, germline-specific overexpression of the wild-type *lmd-3* gene in the mutant background fully rescued progeny counts to wild-type levels (S2E Fig in S1 File). Collectively, these findings indicate that LMD-3 functions cell-autonomously within the germline to sustain early oocyte production.

Having established this germline autonomy, we proceeded to examine the structural integrity of the germline tissue [31]. The *C. elegans* germline is a dynamic, spatially organized tissue defined by distinct zones of proliferation, meiosis, and differentiation, all of which are critical for sustainable oogenesis (S2A Fig in S1 File). Germ cell pools are maintained by a distal stem cell region regulated by the distal tip cell (DTC), providing a clear spatial framework for visualizing germ cell progression throughout the gonad [32].

Quantitative analysis of DAPI-stained gonads confirmed a significant reduction in the total number of germ cells in Day 1 adult *lmd-3*^W690C^ mutants (Fig 2C and 2D). This defect was accompanied by a marked decline in the number of proliferative germline stem cells (Fig 2E), suggesting that the mutation fundamentally compromises the maintenance of the progenitor pool required for ongoing germ cell production and homeostasis [33].

To elucidate the downstream mechanism driving this germ cell loss, we quantified germline apoptosis, a stress-responsive process frequently linked to compromised oocyte quality [34]. We observed a significant elevation in apoptotic cell corpses within the germlines of *lmd-3*^W690C^ mutants at Day 1 and Day 2 of adulthood (Fig 2F, 2G; S2F and S2G Fig in S1 File). These findings demonstrate a robust correlation between increased germ cell death and reduced fecundity in this POI model. Our results collectively show that the *lmd-3*^W690C^ mutation compromises germline integrity and reproductive capacity, characterized by impaired stem cell maintenance and elevated apoptosis.

### The LMD-3 W690C mutation induces widespread and chronic cellular stress

To characterize the physiological implications of the W690C substitution, we first analyzed the expression profile and subcellular localization of LMD-3. An integrated (Is) transcriptional reporter demonstrated a broad tissue distribution of *lmd-3*, including the nervous system, intestine, and reproductive tract (Fig 3A) [27]. Subcellular fractionation confirmed that N-terminal mCherry-tagged LMD-3, including its TLDc domain, resides primarily within the cytoplasmic and membrane fractions (Fig 3B), consistent with its established lysosomal and V-ATPase-associated functions [22,23]. We also identified a minor nuclear localized fraction, which aligns with the hypothesized transcriptional and antioxidant roles attributed to its human NCOA7 and OXR1 orthologs (Fig 3B) [35,36].

**Fig 3:**
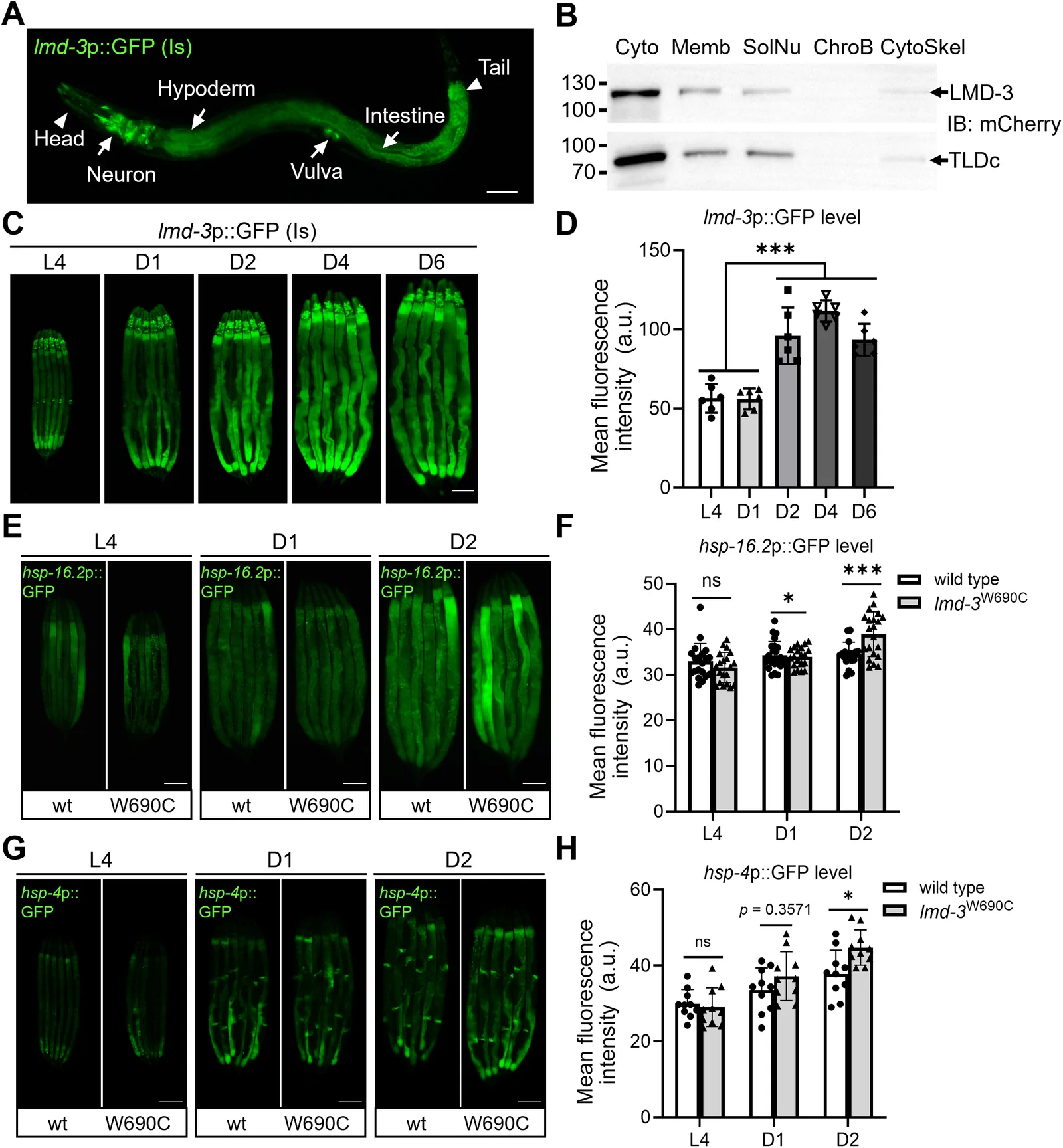
LMD-3 expression pattern and the impact of the W690C mutation on stress responses. (A) Expression pattern of *lmd-3* at the L4 larval stage. Representative images show the expression of the *lmd-3p*::GFP transcriptional reporter. Is, integrated transgene. Scale bars, 100 µm. (B) Subcellular localization of full-length LMD-3 and its TLDc domain. The distribution of mCherry-tagged full-length LMD-3 (upper) and mCherry-tagged TLDc domain (lower) was analyzed. The fractions analyzed were cytoplasmic (Cyto), membrane (Memb), soluble nuclear (SolNu), chromatin-bound (ChroB), and cytoskeletal (CytoSkel). (C, D) Transcriptional level of *lmd-3* during different developmental stages with representative fluorescence images (C) and mean fluorescence intensity quantification (D) using the *lmd-3*p::GFP reporters (n = 6 for each group, ***p < 0.001, with effect sizes relative to L4 being Cohen’s *d* = 0.046 at D1, Cohen’s *d* = 2.788 at D2, Cohen’s *d* = 6.934 at D4, and Cohen’s *d* = 3.827 at D6). Is, integrated transgene. Scale bars, 100 μm. a.u., arbitrary units. (E, F) Representative fluorescence images (E) and mean fluorescence intensity quantification (F) of the UPR^cyto^ reporter *hsp-16.2*p::GFP in wild type and *lmd-3*^W690C^ animals at indicated stages (n ≥ 20 for each group, ns, no significant difference with Cohen’s *d* = 0.368, *p < 0.05 with Cohen’s *d* = 0.169, ***p < 0.001 with Cohen’s d = 1.117). Scale bars represent 100 μm. a.u., arbitrary units. L4, the fourth larval stage. D1, Day 1 of adulthood. D2, Day 2 of adulthood. (G, H) Representative fluorescence images (G) and mean fluorescence intensity quantification (H) of the UPR^ER^ reporter *hsp-4*p::GFP in wild-type and *lmd-3*^W690C^ animals at indicated stages (n = 10 for each group, unpaired t-tests: ns, no significant difference with Cohen’s *d* = 0.207 at L4, and Cohen’s *d* = 0.597 at D1; *p < 0.05 with Cohen’s *d* = 1.259). Scale bars represent 100 μm. a.u., arbitrary units.

Given the established link between oxidative stress and POI [19], and the fact that LMD-3 orthologs exhibit stress-inducible behavior in yeast and humans we investigated whether *lmd-3* expression serves as a protective mechanism during aging. We observed a progressive, age-dependent increase in *lmd-3* transcription (Fig 3C and 3D).

To evaluate whether the W690C mutation impairs global stress resilience, we monitored systemic cytoplasmic and endoplasmic reticulum (ER) stress from the late larval to early adult stages. The cytosolic unfolded protein response (UPR^cyto^), tracked via the *hsp-16.2*p::GFP reporter [37], showed a gradual yet distinct induction in the mutants (Fig 3E, 3F and S2 Table in S1 File), signifying elevated systemic oxidative stress. Concurrently, the ER unfolded protein response (UPR^ER^), visualized by the *hsp-4*p::GFP reporter [38], exhibited a progressive activation profile (Fig 3G, 3H and S2 Table in S1 File).

These findings indicate that the POI-associated W690C mutation triggers widespread, chronic cellular stress, evidenced by the activation of both cytosolic and ER stress pathways. Notably, at the onset of adulthood (Day 1), a time point marked by severe lysosomal dysfunction and reproductive decline, ER stress remains relatively stable, while cytosolic stress is only modestly elevated.

### The W690C-associated lysosomal dysfunction correlates with proteostasis collapse

Building on the established interaction between the TLDc domain and V-ATPases [22,23], and given the central role of lysosomes in aging, we investigated the impact of the W690C mutation on general lysosomal function. Transcriptional profiling of whole-animal lysates revealed a significant downregulation of a broad spectrum of lysosome-related genes in the mutant (Fig 4A). This signature included key components required for lysosomal acidification (V-ATPases), structural integrity (LMP-2, NCR-1), and proteolysis (cathepsin proteases ASP-3, ASP-4).

**Fig 4:**
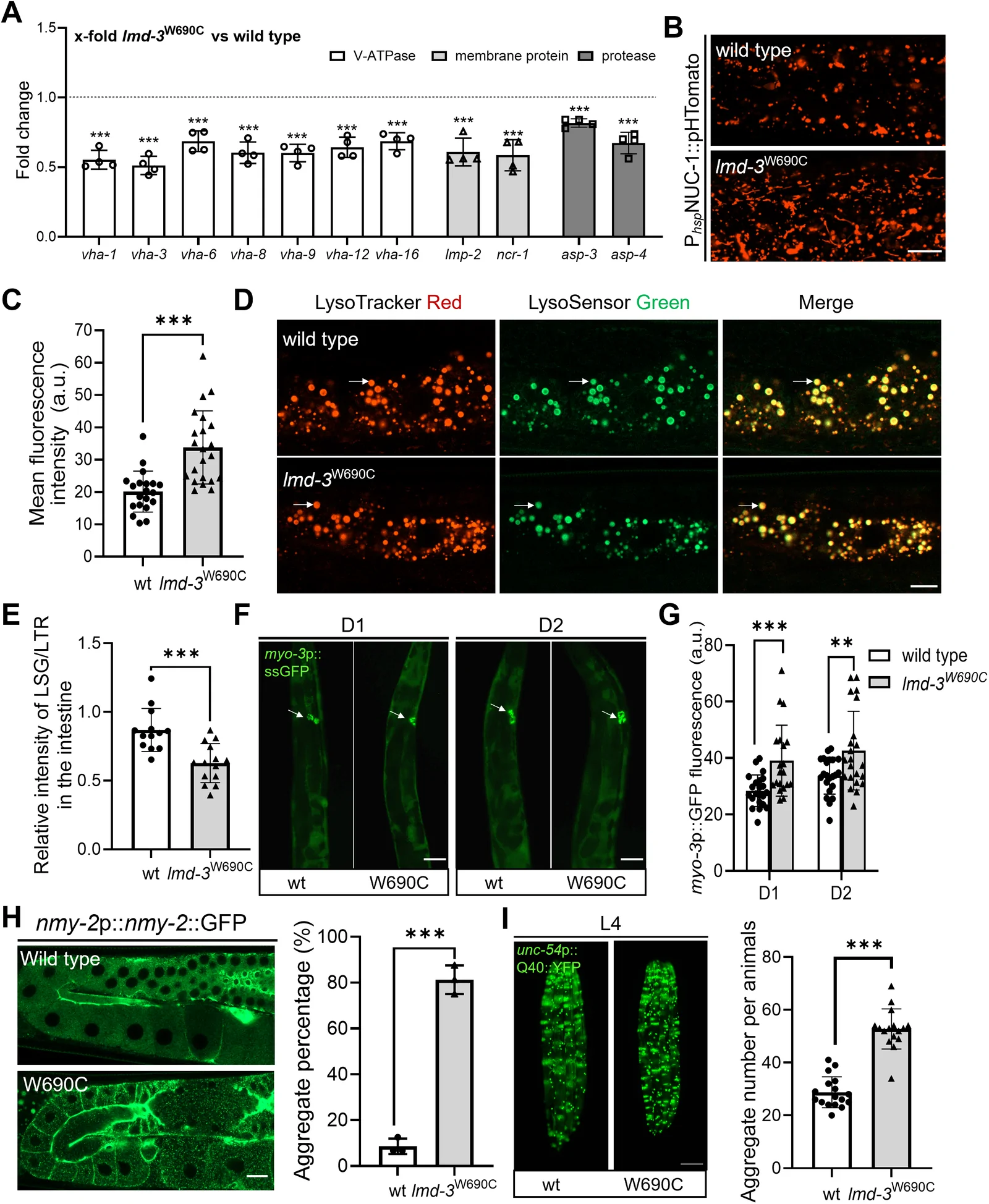
The W690C mutation correlates with proteostasis collapse and lysosomal dysfunction in *C. elegans.* (A) Transcriptional analysis of lysosome-related genes in wild type and *lmd-3*^W690C^ animals at the D1 adult stage (n = 4 independent biological replicates per group, each performed in technical triplicates, unpaired t-tests: ***p < 0.001; genes were analyzed as predefined candidate targets; the complete set of Cohen’s *d* for each gene is provided in S1 Data). (B, C) Representative fluorescence images (B) and mean fluorescence intensity quantification (C) of NUC-1::pHTomato expression in the hypodermis driven by a heat-shock (hs) promoter (n ≥ 20 per group, ***p < 0.001, Cohen’s *d* = 1.477). a.u., arbitrary units. Scale bar, 10 μm. (D, E) Representative confocal fluorescence images (D) and quantification (E) of intestines co-stained with Lysosome-Specific Green (LSG DND-189) and Lysosome-Targeting Red (LTR DND-99) in wild type and *lmd-3*^W690C^ animals at the D1 adult stage. White arrowheads indicate vesicular lysosomes stained by both dyes. Relative intensity of LSG/LTR were quantified (n ≥ 13 per group, ***p < 0.001, Cohen’s *d* = 1.615). Scale bars, 10 μm. (F, G) Representative fluorescence images (F) and mean fluorescence intensity quantification (G) of secreted protein accumulation (*myo-3*p::ssGFP) in the coelomocytes of wild type and *lmd-3*^W690C^ animals at the D1 and D2 stages (n ≥ 20 per group, unpaired t-tests: **p < 0.01, ***p < 0.001, with effect sizes of Cohen’s *d* = 1.047 at D1 and Cohen’s *d* = 0.764 at D2). Scale bars, 50 μm. a.u., arbitrary units. (H) Confocal fluorescence images and quantification of NMY-2::GFP accumulation in wild type and *lmd-3*^W690C^ animals at D1 stage (mean ± SEM, n = 3 biological replicates, n ≥ 12 per condition per replicate, unpaired t-tests: ***p < 0.001, Cohen’s *d* = 14.471). (I) Fluorescence images and quantification of Q40::YFP accumulation in the body wall muscles in wild type and *lmd-3*^W690C^ animals at the L4 stage (n ≥ 16 per group, unpaired t-tests: ***p < 0.001, Cohen’s *d* = 3.539). Scale bars, 50 μm.

To determine whether these transcriptional defects result in a functional loss of lysosomal activity, we assessed lysosomal acidification, which is essential for efficient protein degradation [39]. Analysis using a heat-shock-inducible pH-sensitive reporter, NUC-1::pHTomato [40], revealed a significantly higher fluorescence intensity in the mutant hypodermis, signifying a less acidic, more neutralized intralysosomal pH (Fig 4B, 4C; S3A and S3B Fig in S1 File). This finding was further validated by co-staining with the acidotropic dye LysoSensor Green (LSG) and the lysosome-specific dye LysoTracker Red (LTR) [41]. The reduced LSG/LTR fluorescence ratio in the mutant definitively confirmed that the transcriptional alterations translate into a severe impairment of lysosomal acidity in *C. elegans* (Fig 4D, 4E; S3C and S3D Fig in S1 File).

This systemic acidification defect coincided with a failure in degradative capacity, which we assessed by monitoring the clearance of secreted ssGFP [42]. The reporter exhibited a significant accumulation of fluorescence in the coelomocytes of the mutant (Fig 4F and 4G), demonstrating a block in lysosomal degradation. This defect was associated with a broader collapse in proteostasis, characterized by extensive aggregation of the age-related protein NMY-2::GFP in oocytes [43] (Fig 4H) and the polyglutamine reporter Q40::YFP in body wall muscles [44] (Fig 4I). The vital role of LMD-3 in maintaining protein homeostasis was further underscored by the accumulation of vitellogenin—a primary egg yolk protein—in the mutant’s intestine, pseudocoelom, and germline (S3E Fig in S1 File), confirming that LMD-3 deficiency disrupts the processing of crucial reproductive proteins.

Building upon the broad genetic and biochemical framework established in our recent work [24], genetic compromise V-ATPase activity using the *vha-12(ok821)* loss-of-function allele not only recapitulated the reproductive defects observed in the W690C mutant but also exacerbated the decline (S3F Fig in S1 File). Consistent with the established synergistic network between LMD-3 and V-ATPases [24], these findings support a working model in which LMD-3 functionally interacts with V-ATPase-dependent pathways to maintenance lysosomal homeostasis [6,19,45].

### Vitamin B12 supplementation mitigates LMD-3(W690C)-associated autophagic-lysosomal defects

Building on our findings of systemic lysosomal dysfunction and proteostasis collapse in the *lmd-3*^W690C^ mutant, which exhibits phenotypes reminiscent of the defective autophagy and senescence linked to NCOA7 dysfunction in human POI [19], we next investigated the integrity of the autophagic pathway. We first utilized a transcriptional reporter for *tts-1*, a stress-inducible long non-coding RNA (lncRNA) that represses global translation and serves as a direct transcriptional target of HLH-30/TFEB, the master regulator of autophagy [46]. In contrast to wild-type animals, *lmd-3W690C* mutants exhibited a significant global reduction in *tts-1*p::GFP fluorescence (Fig 5A). This was corroborated by the systemic downregulation of essential autophagy genes, including *hlh-30* [47], and genes required for autophagosome formation, cargo recognition, fusion, and degradation (Fig 5B). Given that HLH-30 activity relies on nuclear translocation, we examined the subcellular localization of HLH-30::GFP; we found that stress-induced nuclear entry of HLH-30 was significantly impaired in *lmd-3*^W690C^ mutants (S4A Fig in S1 File), thereby compromising HLH-30-dependent transcriptional programs. Together, these findings indicate that the W690C mutation severely compromises both the biogenesis and function of the autophagic system.

**Fig 5:**
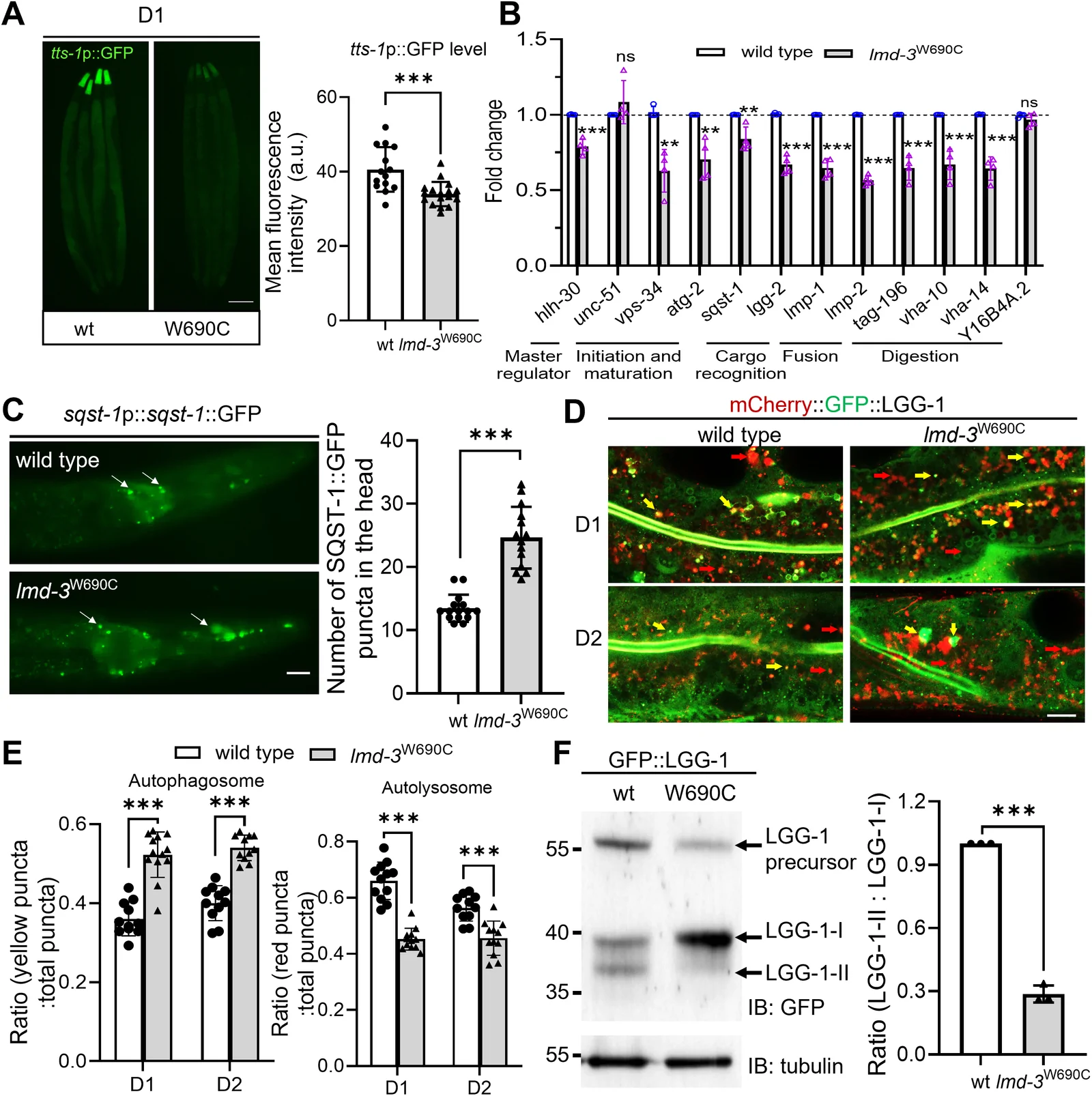
*lmd-3*^W690C^ mutation is associated with impaired autophagy and autophagic flux in *C. elegans.* (A) Representative fluorescence images and mean fluorescence intensity quantification of the autophagy reporter *tts-1*p::GFP in wild type and *lmd-3*^W690C^ animals at the indicated stages (n ≥ 14 per group, ***p < 0.001, Cohen’s *d* = 1.417). a.u., arbitrary units. Scale bars represent 100 μm. (B) Transcriptional analysis of key autophagy genes was performed in wild type and *lmd-3*^W690C^ animals, and gene expression was quantified by RT-qPCR (n = 4 independent biological replicates per group, each performed in technical triplicates, unpaired t-tests: analyzed as predefined candidate targets; **p < 0.01, ***p < 0.001, ns, no significant difference; Cohen’s *d* values for each gene is provided in S1 Data). (C) Representative fluorescence images and quantification of SQST-1::GFP accumulation in wild type and *lmd-3*^W690C^ animals at D1 adult stage (n ≥ 14 per group, ***p < 0.001, Cohen’s *d* = 4.017). White arrowheads denote fluorescent protein aggregates. Scale bars, 50 μm. (D, E) Representative confocal images (D) and quantification (E) of the mCherry::GFP::LGG-1 reporter in wild type and *lmd-3*^W690C^ animals at D1 and D2 adult stages. The ratio of autophagosomes (yellow puncta, yellow arrow) to total puncta and autolysosomes (red puncta, red arrow) to total puncta was quantified (n ≥ 11 per group, ***p < 0.001; Cohen’s *d* values are provided in S1 Data). Scale bar, 10 μm. (F) Representative Western blot and corresponding quantification of the ratio of lipidated LGG-1-II to unlipidated LGG-1-I protein in wild-type and *lmd-3*^W690C^ mutant animals (n = 3 biological replicates, ***p < 0.001, Cohen’s *d* = 24.773). a.u., arbitrary units. IB, immunoblotting.

To confirm that these transcriptional defects correlated with functional impairment, we assessed the accumulation of SQST-1, the *C. elegans* ortholog of p62. Efficient clearance of SQST-1 is indicative of a healthy autophagic flux, and we observed a massive accumulation of SQST-1::GFP aggregates in the adult W690C mutants (Fig 5C), providing strong evidence that the autophagic degradation pathway is severely disrupted. Autophagic flux was assessed using the tandem fluorescent reporter mCherry::GFP::LGG-1 [48]. Compared to wild-type animals, W690C mutants exhibited a dramatic increase in autophagosomes (yellow puncta) and a concomitant decrease in mature autolysosomes (red puncta) (Fig 5D, 5E; S4B and S4C Fig in S1 File). These findings indicate that autophagic flux is impaired at the maturation and degradation stage. Furthermore, Western blot analysis of GFP::LGG-1 in whole-animal lysates revealed a simultaneous defect in autophagosome biogenesis [49], evidenced by a significant reduction in the ratio of membrane-associated LGG-1-II (lipidated) to cytosolic LGG-1-I (unlipidated) (Fig 5F). These findings confirm that the W690C mutation induces a comprehensive, multi-stage failure of the autophagic-lysosomal pathway.

Given that *lmd-3*^W690C^ dysfunction impairs lysosomal activity and reproductive health, we hypothesized that enhancing cellular resilience could restore cellular and reproductive homeostasis. Since lysosomes are critical for the uptake and processing of dietary vitamin B12, the lysosomal failure in *lmd-3* mutants likely induces conditions consistent with reduced vitamin B12 availability [50]. We therefore assessed the capacity of mecobalamin (meCbl), an active form of vitamin B12, to mitigate these defects. Strikingly, treatment with 150 nM meCbl fully restored the autophagosome-to-autolysosome ratio to wild-type levels, indicating rescued autophagic flux (Fig 6A and 6B).

**Fig 6:**
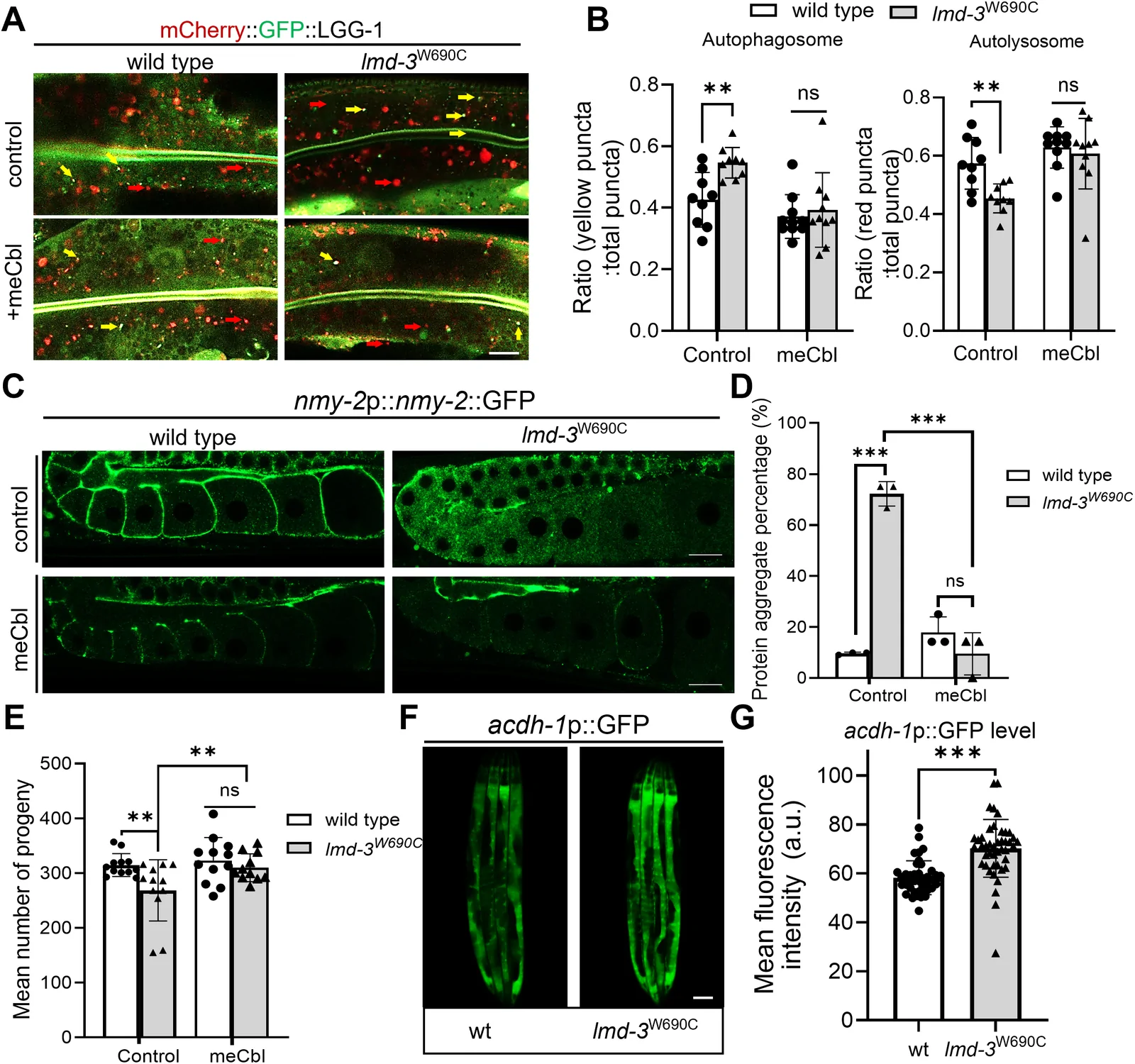
meCbl supplementation restores proteostasis and mitigates autophagic-lysosomal defects in *lmd-3*^W690C^ mutants. (A, B) Representative confocal images (A) and quantification (B) of the mCherry::GFP::LGG-1 reporter in wild type and *lmd-3*^W690C^ animals with mecobalamin (meCbl) supplementation at D1 adult stages. The ratio of autophagosomes (yellow puncta, yellow arrow) to total puncta and autolysosomes (red puncta, red arrow) to total puncta was quantified (n ≥ 9 per group, **p < 0.01, ns, no significant difference; Cohen’s *d* values are provided in S1 Data). Scale bar, 10 μm. (C, D) Confocal fluorescence images (C) and corresponding quantification (D) of *nmy-2*p::*nmy-2*::GFP accumulation in the oocytes of wild-type and *lmd-3*^W690C^ mutants at D1 of adulthood, with mecobalamin (meCbl) supplementation (mean ± SEM, n = 3 biological replicates, animals per condition per replicate ≥ 10, ***p < 0.001, ns, no significant differences; Cohen’s *d* values are provided in S1 Data). Scale bars, 10 μm. (E) Total brood sizes showing that meCbl treatment rescues the reduced fecundity of *lmd-3*^W690C^. Data are presented as mean ± SEM (n ≥ 11 per group, **p < 0.01, ns, no significant difference; Cohen’s *d* values are provided in S1 Data). (F, G) Representative fluorescence images (F) and mean fluorescence intensity quantification (G) of the B12 reporter *acdh-1*p::GFP in wild type and *lmd-3*^W690C^ animals at the indicated stages (n ≥ 40 per group, ***p < 0.001, Cohen’s *d* = 1.230). a.u., arbitrary units. Scale bars represent 100 μm.

Consistent with this recovery, meCbl supplementation significantly reduced NMY-2::GFP aggregates in the germline (Fig 6C and 6D) and enhanced ssGFP clearance in coelomocytes (S5A, S5B Fig in S1 File). Crucially, meCbl treatment restored the diminished brood size of *lmd-3*^W690C^ mutants to wild-type levels (Fig 6E). Finally, using a dietary B12 sensor strain carrying an *acdh‑1*p::GFP reporter [51], a marker for the B12-independent propionate shunt pathway [52], we observed significant activation of *acdh-1* transcription in *lmd-3*^W690C^ animals (Fig 6F and 6G), strongly indicating that the W690C mutation is consistent with impaired B12-dependent metabolism.

### The W690C mutation impairs LMD-3 expression and structural integrity

Having established the link between the W690C mutation and profound systemic lysosomal and reproductive dysfunction, we sought to delineate the biochemical consequences of this specific point mutation—a key distinction from previous complete loss-of-function models. We first investigated whether the mutation affected *lmd-3* transcript level. Transcriptional analysis across three distinct regions of the *lmd-3* gene (Fig 7A and S3 Table) revealed a significant and consistent reduction in overall *lmd-3* transcript levels in the mutant compared to wild-type animals (Fig 7B), indicating that the mutation results in reduced steady-state transcript levels.

**Fig 7:**
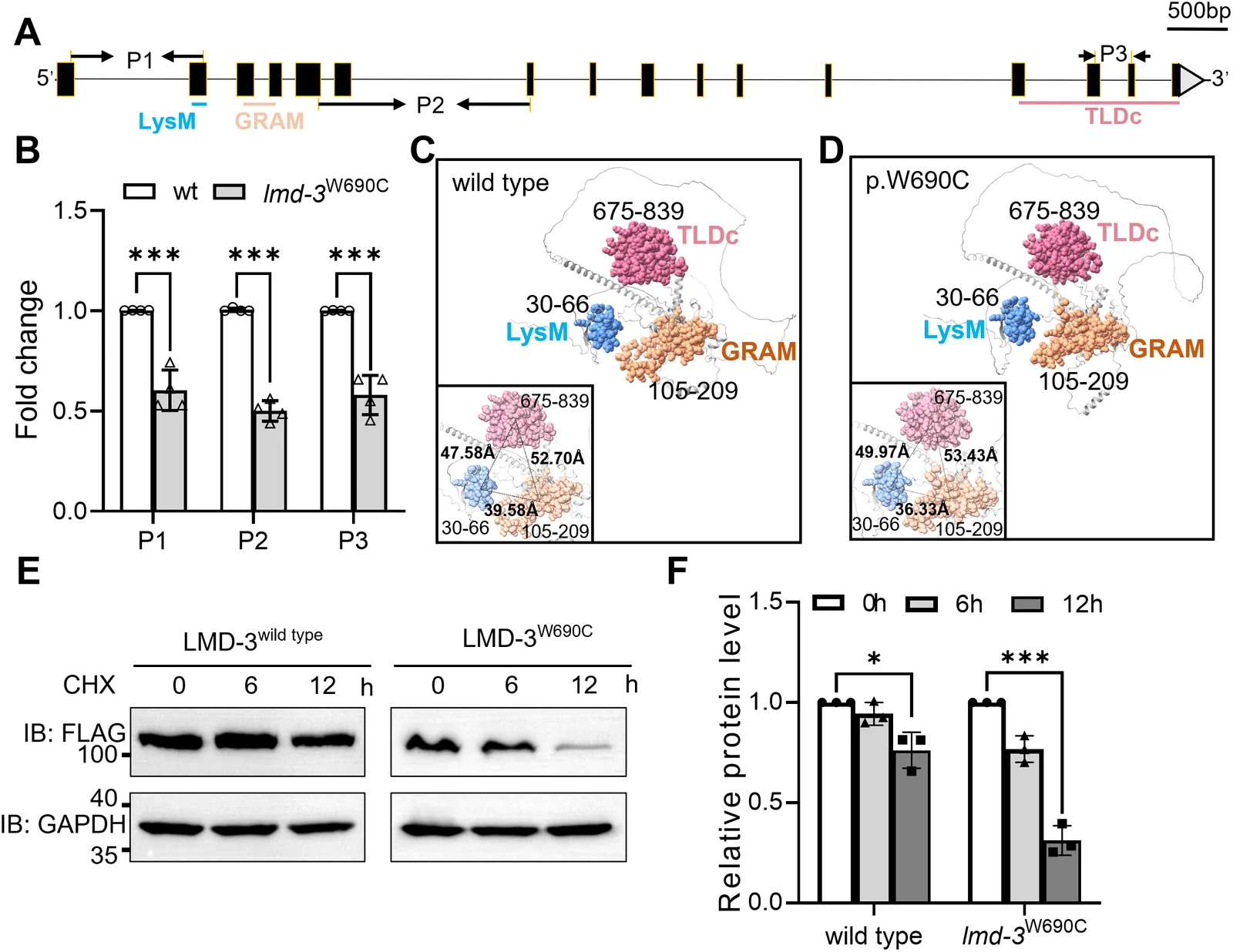
The W690C mutation compromises LMD-3 protein structure and stability. (A) The schematic diagram shows the genomic structure of *lmd-3*, including the positions of its conserved LysM, GRAM, and TLDc domains. The locations of the qRT-PCR primers (P1, P2, P3) designed to quantify *lmd-3* mRNA are also shown. (B) qRT-PCR measurements for endogenous *lmd-3* mRNA levels in wild type (wt) and *lmd-3*^W690C^ animals using primer sets P1, P2, and P3 (n = 4 independent biological replicates per group, each performed in technical triplicates, unpaired t-tests: ***p < 0.001, with effect sizes of Cohen’s *d* = 5.548 for P1, Cohen’s *d* = 13.762 for P2, and Cohen’s *d* = 6.039 for P3)*.* (C, D) Structural analysis of the LMD-3^W690C^ protein. Predicted three-dimensional structures of wild-type (C) and W690C (D) proteins are presented to illustrate the effects of the W690C mutation on protein folding. Key domains are color-coded: LysM (cornflower blue), GRAM (light salmon), and TLDc (pale violet red). A magnified view shows the trimer-like region, where the mutation leads to altered inter-subunit distances in the p.W690C protein (49.97 Å, 53.43 Å, 36.33 Å) compared to the wild-type (47.58 Å, 52.70 Å, 39.58 Å). (E, F) The W690C mutation reduces LMD-3 protein stability. Representative Western blot images (E) and semi-quantitative analysis (F) show the turnover of 1xFLAG-tagged LMD-3 in HEK-293T cells treated with cycloheximide (CHX) for 0, 6, and 12 h (n = 3 biological replicates, *p < 0.05, ***p < 0.001, with effect sizes for 0 vs 12 h being Cohen’s *d* = 3.740 for wild type and Cohen’s *d* = 13.122 for *lmd-3*^W690C^). a.u., arbitrary units.

Given the established link between human POI-associated NCOA7 mutations and protein stability [19], we reasoned that the W690C mutation might also destabilize the LMD-3 protein. Based on three-dimensional structural modeling of *C. elegans* wild-type and W690C-mutated LMD-3 [53], LMD-3 is predicted to form a conserved trimer-like assembly comprising LysM, GRAM, and TLDc domains (Fig 7C). While the overall core conformation remained largely preserved, the W690C mutation significantly altered the inter-subunit distances within the predicted trimer interface (Fig 7D), suggesting a disruption of structural integrity. Complementary analysis using six machine learning-based algorithms corroborated that the W690C mutation compromises overall protein stability (S4 Table in S1 File) [54–58], consistent with findings in human orthologs [19].

Evaluation of LMD-3 protein turnover in a heterologous expression system revealed that while wild-type LMD-3 protein remained relatively stable, the mutant protein exhibited a significant decrease in steady-state levels, showing marked degradation following 12 hours of CHX treatment (Fig 7E, 7F and S6A Fig in S1 File). This provides supportive biochemical evidence that the W690C missense mutation fundamentally compromises intrinsic LMD-3 protein stability, mechanistically linking the structural defect to rapid degradation.

Finally, we confirmed that RNAi knockdown of *lmd-3* in wild-type animals phenocopied all observed defects, including reduced brood size, elevated ER stress, and impaired lysosomal degradation, indicating that the W690C mutation acts primarily through a loss of LMD-3 function rather than gain-of-function or dominant-negative effects (S6B–S6E Fig in S1 File). Collectively, these findings establish that the POI-associated W690C mutation severely impairs both steady-state *lmd-3* transcript levels and protein stability.

## Discussion

Reproductive longevity is a fundamental pillar of healthy aging, yet the molecular basis of premature ovarian insufficiency (POI) remains largely elusive. Recent translational and in vivo evaluations emphasize that preserving ovarian follicular reserve and mitigating cellular stress are critical for combating POI-associated reproductive decline [59]. Our previous work established LMD-3 as a critical regulator of reproductive capacity in *C. elegans* [24]*.* Building upon this foundation, the current study leverages a novel POI-associated W690C knock-in model. This approach allows us to dissect the pathophysiological mechanisms of fertility decline at single-amino-acid resolution. We demonstrate that this pathogenic lesion compromises reproductive health not merely through a simple loss-of-function, but by triggering a profound collapse in cellular proteostasis and lysosomal integrity, which can be partially rescued by vitamin B12 supplementation.

A core finding of our work is the mechanistic distinction between the W690C mutation and complete *lmd-3* loss. Our data reveal that W690C compromises the intrinsic stability of the LMD-3 protein (based on heterologous data) and concurrently reduces its mRNA levels (Fig 7B). This observation contrasts with the human *NCOA7(W804C)* variant, where transcript levels remain stable [19]. The exact mechanism underlying this mRNA reduction in *C. elegans* requires further investigation, and may involve transcript-level destabilization or surveillance pathways. Nevertheless, such species-specific variations in mRNA regulation are widely documented in comparative biology [60]. Furthermore, at the functional level, the existence of the mammalian ortholog *OXR1* may provide a functional buffer that masks the physiological impact of NCOA7 destabilization. By contrast, the non-redundant nature of the *C. elegans lmd-3* locus renders the organism uniquely sensitive to the protein-level destabilization caused by *W690C*, making it an ideal system to map the core pathogenic consequences of this specific mutation.

The structural and functional destabilization of LMD-3(W690C) precipitates systemic lysosomal dysfunction, characterized by defective acidification and impaired degradative capacity. These results align with the evolving concept of the lysosome as a central signaling hub whose failure is intrinsically linked to aging and proteotoxicity [61–63]. We provide evidence that this organelle failure creates a detrimental environment for non-dividing cells like oocytes [6,64], leading to increased germline apoptosis and depletion of the progenitor pool. By linking impaired proteostasis, a hallmark of systemic aging [65], to early-onset ovarian dysfunction, our model bridges the gap between general cellular quality control and specific reproductive health.

Beyond mapping the pathogenic cascade, our study introduces a pioneering therapeutic perspective. Through experimental demonstration, we show that the *lmd-3*^W690C^ mutation causes primary lysosomal and autophagic dysfunction, which correlates with metabolic alterations consistent with reduced vitamin B12 availability and impaired propionate metabolism [66]. Given that methylmalonyl-CoA mutase requires cobalamin to prevent the accumulation of toxic propionate metabolites [67], we propose a working mechanistic model wherein impaired B12-dependent metabolism acts as a significant compounding factor alongside primary proteostatic collapse, rather than a strict linear sequence where lysosomal failure exclusively causes B12 deficiency which subsequently drives all downstream defects.

Within this conceptual framework, supplementation with mecobalamin (meCbl) effectively bypassed the lysosomal-metabolic block, restoring autophagic flux, clearing protein aggregates, and rescuing reproductive output. Parallel studies on metabolic and tissue-protective interventions similarly highlight the efficacy of targeted restorative strategies in rescuing compromised physiological and endocrine functions under stress conditions [68]. This finding underscores the potential for simple nutritional interventions to mitigate the cellular consequences of specific genetic lesions in *C. elegans* reproductive tissues, though its direct clinical applicability to human POI requires rigorous evaluation in mammalian models and clinical settings.

Despite its utility, our model has several limitations when extrapolating to human POI. First, the continuously proliferating syncytial nematode germline contrasts fundamentally with the finite human primordial follicle pool [1,32,33]. Second, mammalian ovarian aging is driven by oocyte exhaustion, whereas nematode reproductive decline involves broader systemic aging factors [28,29]. Third, our systemic assays lack the resolution to model cell-type-specific microenvironments, such as granulosa-oocyte interactions [5,19]. Finally, we rely on the *acdh-1* biosensor to infer impaired B12-dependent metabolism [51,52]; future direct biochemical quantification is required to confirm these metabolic shifts. Nevertheless, our model captures conserved core mechanisms of lysosomal collapse, offering metabolic insights that warrant mammalian validation.

Looking forward, the conservation of the TLDc domain and its V-ATPase-interacting interface suggests that the LMD-3/NCOA7 pathway is a promising target for broad genetic screening in idiopathic POI. Furthermore, our findings open new questions regarding whether the natural decline of wild-type LMD-3/NCOA7 expression contributes to age-related oocyte quality decline [19]. The high-throughput nature of our *C. elegans* model facilitates the screening of compounds that modulate TFEB-mediated lysosomal activity or autophagic flux [48,69,70], potentially expanding the therapeutic landscape for reproductive longevity.

In summary, this study proposes a working mechanistic model linking a clinical POI-associated mutation to intrinsic protein instability, systemic lysosomal failure, and functionally associated alterations in B12-dependent metabolism. By distinguishing our experimentally demonstrated phenotypes from this broader working framework, we validate our *C. elegans* model as a platform for mechanistic discovery. Furthermore, we highlight vitamin B12 as a valuable conceptual framework for understanding and potentially managing early-onset reproductive decline, pending future translational studies.

## Materials and methods

### *C. elegans* strains and maintenance

All *C. elegans* strains (S1 Table), including the wild-type N2 Bristol isolate, were reared at 20 °C on standard nematode growth medium (NGM) plates. Bacterial lawns were established by seeding plates with *Escherichia coli* OP50. To ensure synchronized development for experiments, gravid adults were treated with a standard bleaching solution to harvest eggs.

### CRISPR-Cas9 mediated mutagenesis

The *lmd-3*^W690C^ missense mutation was engineered by SunyBiotech using CRISPR-Cas9 technology. Sanger sequencing was employed to verify the specific point mutation (S3 Table). To minimize potential off-target effects and ensure genetic purity, the resulting mutant line was backcrossed three times against the wild-type N2 strain prior to all downstream phenotypic characterizations.

### Plasmid construction

To enable biochemical assessment in mammalian systems, we developed 1xFLAG-tagged LMD-3 expression constructs. PCR was used to amplify the full-length *lmd-3* cDNA sequences from both wild-type and *lmd-3*^W690C^ mutant backgrounds. These products were subsequently cloned into the pcDNA3.1 vector. Primer sequences are listed (S3 Table).

### Cross-fertilization assay

To assess sperm functionality, cross-fertilization assays were conducted utilizing the spermless *fem-1(hc17)* mutant strain. Synchronized L4 *fem-1(hc17)* hermaphrodites were co-cultured with either wild-type or *lmd-3*^W690C^ males at a 1:4 ratio on 35 mm NGM plates at the restrictive temperature of 25 °C. Following a 20-hour mating period, the hermaphrodites were individually transferred to fresh plates at the same temperature, and the total number of cross-progeny was subsequently quantified.

### Transgene generation and germline rescue

For germline-specific rescue experiments, the *pie-1*p::mCherry::*lmd-3* expression plasmid was introduced into the *lmd-3*^W690C^ mutant background via standard microinjection techniques. The injection mixture comprised the rescue construct, 200 ng/μL of sheared salmon sperm DNA to mitigate transgene silencing, and 5 ng/μL of a *myo-2*p::Tomato Red co-injection marker. Transgenic F1 progeny, identified by red fluorescence in the pharynx, were subsequently isolated for brood size evaluations.

### Quantitative real-time PCR (qRT-PCR)

For transcript quantification, 200 age-synchronized Day 1 adults were collected in M9 buffer, subjected to lysis using Trizol Reagent (Invitrogen, 15596026CN), and flash-frozen in liquid nitrogen. Total RNA was isolated through chloroform/isopropanol extraction. Subsequently, 1.2 μg of RNA was reverse-transcribed into cDNA using the HiScript III 1st Strand cDNA Synthesis Kit (+ gDNA wiper) (Vazyme, R312-01). qRT-PCR was conducted on an ABI QuantStudio 1 system (Thermo Fisher Scientific) utilizing ChamQ SYBR Color qPCR Master Mix (Vazyme, Q411-02). Data were normalized to the *act-1* endogenous control.

### Reproductive capacity analysis

In accordance with established protocols [71], a minimum of 10 synchronized L4 hermaphrodites per strain were individually isolated on fresh NGM plates. Worms were transferred to new plates every 12 hours until the cessation of egg-laying. The total progeny output per individual was determined by counting the offspring collected from each transfer. Experiments were executed in three biological replicates.

### Measurement of body length

Worms at specified developmental stages were anesthetized in levamisole (Sangon Biotech, China) and immobilized on agarose pads. Bright-field images were captured via a Nexcope 910 microscopy system. Body length (head-to-tail distance) was calculated using Image View software, with a minimum of 22 animals analyzed per experimental cohort.

### Microscopy and imaging analysis

For all fluorescence and bright-field imaging, animals were anesthetized with levamisole and mounted on agarose pads. Depending on the resolution requirements, images were acquired using either a Nexcope 910 fluorescence microscope or a Zeiss LSM 980 confocal microscope. Quantitative image metrics, such as fluorescence intensity and puncta characteristics, were assessed using ImageJ (1.54J, NIH). For fluorescence intensity, the mean signal (total intensity per region of interest) was recorded. Growth-related variation was mitigated by normalizing intensity to the animal’s body area. All quantification was conducted by researchers blinded to the experimental genotype to prevent observer bias.

### Germline staining and quantification

To assess germline integrity, synchronized Day 1 adults were fixed in ice-cold methanol for 5 minutes, followed by three washes in M9 buffer. Nuclei were visualized by incubating samples in DAPI (Macklin, D807022) for 30 minutes in the dark, followed by additional washing steps. Confocal microscopy was used to examine the gonads. The total germ cell population and proliferative stem cell counts were determined quantitatively using ImageJ (1.54J, NIH).

### SYTO12 staining for germline apoptotic cells

Synchronized larvae were reared to Day 1 and Day 2 of adulthood. Animals were incubated in SYTO 12 dye (Thermo Fisher Scientific, S7575) for 3 hours in the dark. Post-incubation, worms underwent three washes via centrifugation (4,000 rpm, 1 minute) and were allowed a 1-hour recovery period on NGM plates to clear residual dye from the gut. Anesthetized animals were examined via green fluorescence microscopy to score apoptotic corpses.

### Dual lysosomal staining and quantification

As previously described [72], synchronized Day 1 adults were co-incubated with 10 mM LysoSensor Green DND-189 and LysoTracker Red DND-99 (Yeasen) for 1 hour at 20 °C in the dark. A 1-hour recovery phase on OP50-seeded NGM plates followed. Lysosomal acidity was assessed by calculating the fluorescence ratio of LysoSensor Green to LysoTracker Red via ImageJ (1.54J, NIH); a reduced ratio indicates diminished lysosomal acidity.

### Cell culture and Transient transfection

HEK-293T cells were cultured in DMEM (C119955-00BT, Grand Island Biological Company) containing 10% fetal bovine serum (Excell Media) at 37 °C in a 5% CO^2^ atmosphere. Cells were transfected using polyethyleneimine (PEI) (Sigma-Aldrich, 408727) at a DNA-to-PEI ratio of 1:4.5, with 1 µg of total DNA used per 10^6 cells.

### Cycloheximide pulse chase assay

Protein turnover was evaluated via a Cycloheximide (CHX) chase assay [73]. Thirty-six hours post-transfection, cells were treated with 50 µg/mL CHX to inhibit new protein synthesis. At specified intervals, cells were harvested to quantify protein levels via Western blot analysis.

### RNA interference (RNAi)

RNAi experiments were conducted utilizing an RNAi-competent *E. coli* OP50 strain (iOP50) transformed with either the L4440 empty vector (control) or an *lmd-3* RNAi construct. iOP50 cultures were grown in LB broth containing 100 µg/mL ampicillin, then seeded onto NGM plates supplemented with 100 µg/mL ampicillin and 1 mM IPTG to initiate dsRNA production. Plates were incubated overnight at 25 °C. Synchronized L4 larvae were transferred to these plates and maintained at 20 °C until they reached the young adult stage for downstream analysis.

### Autophagy flux and aggregation analysis

Autophagic flux was monitored using the tandem reporter mCherry::GFP::LGG-1. In anterior intestinal cells, autophagosomes (AP) were identified as GFP/mCherry-positive (yellow) puncta, while autolysosomes (AL) were identified as mCherry-only (red) puncta (due to GFP quenching). SQST-1::GFP aggregates in head neurons were similarly quantified. To ensure stringency, quantification was performed manually by two independent, blinded investigators, and results were averaged. A minimum of 11 worms per condition were analyzed across three biological replicates.

### Mecobalamin supplementation

A 150 µM stock of meCbl (Macklin, M812748) was dissolved in H_2_O (also used for control plates). For treatment, the stock was diluted into the NGM agar medium to a final concentration of 0.15 µM prior to plate pouring, ensuring continuous exposure throughout the lifecycle.

### Quantification of *myo-3*p::ssGFP intensity

Day 1 adult *C. elegans* expressing the secreted *myo-3*p::ssGFP reporter were imaged. Coelomocytes were manually identified as regions of interest (ROI) using ImageJ (1.54J, NIH). Mean fluorescence intensity was calculated for each ROI, and values from all coelomocytes in an individual animal were averaged. Background fluorescence from non-coelomocyte regions was subtracted. At least 18 animals were evaluated per group.

### Quantification of NMY-2::GFP aggregates in germline oocytes

Day 1 adults expressing NMY-2::GFP were selected for evaluation. Using a Zeiss LSM 980 confocal microscope, we determined the presence or absence of aggregates in the two most proximal oocytes of the gonad. Scoring was performed by a blinded investigator. The aggregate proportion was calculated from three independent biological replicates, with at least 10 animals analyzed per replicate.

### Quantification of NUC-1::pHTomato intensity

Lysosomal acidification was assessed using the pH-sensitive reporter P*hs*NUC-1::pHTomato [40,43]. Expression was induced by a 30-minute heat shock at 33 °C, followed by a 24-hour recovery at 20 °C. Mean pHTomato fluorescence intensity per unit area in the hypodermis was quantified using ImageJ (1.54J, NIH) in at least 20 animals per strain.

### Western blotting

Day 1 adults (n = 200) were lysed in 1 × sample buffer and boiled at 95 °C for 10 minutes. Protein extracts were separated via SDS-PAGE and transferred to PVDF membranes. Membranes were blocked with 5% non-fat milk in TBST and probed with primary antibodies: anti-GFP (1:1000, abclonal, AE011), anti-α-tubulin (1:5000, Sigma-Aldrich, T5168), anti-flag DDDDK-tag (1:10000, MBL, M185), and anti-GAPDH (1:500, Absin, abs830030ss). Signals were detected using ECL (Tanon 5200). Due to stripping constraints, parallel gels were used for target proteins and loading controls. Raw, uncropped blot data are included (S2 Data).

### Protein sequence and structure prediction

Human NCOA7 (NP_001186548.1) and *C. elegans* LMD-3 (NP_505173.2) sequences were retrieved from NCBI. 3D modeling was performed using AlphaFold3 [53]. Predicted structures were analyzed via ChimeraX 1.10.1 (University of California, San Francisco).

### Inter-domain distance measurement

To quantify the structural impact of the mutation on the predicted LMD-3 trimer-like region, the distances between the conserved LysM, GRAM, and TLDc domains were measured. The centroid of each respective domain was defined as a vertex of a triangle. The distances between these centroids were then calculated using the “Distance” function within the “Annotation” module of ChimeraX 1.10.1.

### Protein stability prediction

To assess the effect of the point mutation on protein stability, we employed six distinct machine learning-based prediction tools [54–58]. The LMD-3 protein sequence and 3D structural model were the same as those used for the structural analysis above. For each web-based tool, the W690C substitution was specified to calculate the change in free energy (ΔΔG). The definition of a “Destabilizing” prediction varied among the different websites, corresponding to specific ΔΔG thresholds.

### Statistical analysis

All data were statistically analyzed using GraphPad Prism 9 (La Jolla, CA, USA). Data are presented as the mean, with error bars representing the standard error of the mean (SEM). Student’s t-tests were utilized for comparisons between two distinct groups, while one-way ANOVA tests were performed for multiple group comparisons. To evaluate the magnitude of differences between groups, effect sizes were calculated using Cohen’s *d* where appropriate using SPSS 27 (Chicago, USA). Statistical significance was established at *p* < 0.05 (indicated by *), *p* < 0.01 (indicated by **), and *p* < 0.001(indicated by ***); ns, no significant difference. For targeted transcript analyses (such as lysosomal and autophagy-related genes in Figs 4A and 5B), the evaluated genes were treated as predefined candidate targets and assessed using targeted individual hypothesis tests without multiple testing corrections.

## Supporting information

S1 File Supplementary Figures and Tables. This file contains S1 to S6 Figs, S2 Table, and S4 Table.
(PDF)

S1 Video Structural conservation of the TLDc domains. This movie presents a 360-degree rotation of the aligned TLDc domains from human NCOA7 (pink) and *C. elegans* LMD-3 (light blue). The movie highlights the remarkable structural conservation, with key conserved tryptophan residues (human W804 and *C. elegans* W690) highlighted by black arrows and colored bright green and yellow, respectively.
(MP4)

S1 Table *C. elegans* strains used in this study. (XLSX)

S3 Table Primers and oligos used in this study. (XLSX)

S1 Data Comprehensive record of experimental raw data. (XLSX)

S2 Data Uncropped scans of western blot membranes. (DOCX)
