# Supplementary material for: A premature ovarian insufficiency-associated LMD-3 point mutation impairs protein stability and causes autophagic-lysosomal dysfunction in Caenorhabditis elegans
Source: PLoS One. 2026 Oct 27;21(10):e0361215. doi: 10.1371/journal.pone.0361215 (PMC13712518; doi:10.1371/journal.pone.0361215)
Supplement: Supplementary file 1 [file pone.0361215.s001.pdf]

Supporting information: S1 File. Supplementary Figures and Tables.

This PDF file includes:

S1 Fig. Sequence alignment highlighting conserved domains and the POI-associated residue.

S2 Fig. The *lmd-3*^{W690C} mutation in *C. elegans* is associated with impaired fecundity and increased germline apoptosis.

S3 Fig. The W690C mutation correlates with impaired protein degradation and lysosomal function.

S4 Fig. *lmd-3*^{W690C} mutation is associated with impaired autophagic transcription and autophagic flux.

S5 Fig. Mecobalamin rescues secreted protein accumulation defects in *lmd-3*^{W690C} mutants.

S6 Fig. The W690C mutation reduces *lmd-3* transcript levels and correlates with compromised protein stability.

S2 Table. Summary of examined reporters and associated germline defects in *lmd-3*^{W690C} mutants.

S4 Table. Machine learning-based prediction of LMD-3^{W690C} stability.

Other Supplementary Materials for this manuscript include the following:

S1 Video. Structural conservation of the TLDc domains. (Data provided in a separate .mp4 format file)

S1 Table. *C. elegans* strains used in this study. (Data provided in a separate Excel spreadsheet)

S3 Table. Primers and oligos used in this study. (Data provided in a separate Excel spreadsheet)

S1 Data. Comprehensive record of experimental raw data. (Data provided in a separate Excel spreadsheet)

S2 Data. Uncropped scans of western blot membranes. (Images provided in a separate Word document)

S1 to S6 Figs

S1 Fig

```
LMD-3   0 ---------------------------------------------------------------------- 0
NCOA7   1 MDTKEEKKERKQSYFARLKKKKQAKQNAETASAVATRTHTGKEDNNTVVLEPDKCNIAVEEEYMTDEKKK 70
                                Q23X
                                                             LysM
LMD-3   0 -------------MGSFGSVSS---------VPPGSAKIPAFQMDYTITETDTLERVAASHDCTVGELMK 48
NCOA7  71 RKSNQLKEIRRTELKRYYSIDDNQNKTHDKKEKKMVVQKPHGTMEYTAGNQDTLNSIALKFNITPNKLVE 140
                       :  : *:..              .: *   *:**  : ***: :* ..: * .:*::
                                                                   A128T

LMD-3  49 LNKMASRMVFPGQKILVPLANADDVFDPPSSASSLKSSGNQISGIENAEDGIRKGPGGAVPAHVRRGSFT 103
NCOA7 141 LNKLFTHTIVPGQVLFVPDANSPSSTLRLSSS----SPGATVSPS-SSDAEYDKLP---------DADLA 196
          ***: :: :.*** ::** **: .     **:    * *  :*   .::    * *          ..::
             T146I
                                                            GRAM
LMD-3 104 KTQSAPIPR--GSNDEADTDCLQRFLKIKVKQVTESDGTVSGTLLVTPNCLMFDPDVSHPLVKENGPDLY 186
NCOA7 197 RKALKPIERVLSSTSEEDEPGVVKFLKMNCRYFTDGKGVVGGVMIVTPNNIMFDPHKSDPLVIENGCEEY 266
          :.   ** *  .*..* *   : :***:: : .*:..*.*.*.::**** :****. *.*** *** : *

LMD-3 187 GMVANMDEIQSVSVYKEISGLTGDKAEK------KRDIFDPDHLRTPED----SP--------KKQIKQN 238
NCOA7 267 GLICPMEEVVSIALYNDISHMKIKDALPSDLPQDLCPLYRPGEWEDLASEKDINPFSKFKSINKEKRQQN 336
          *::. *:*: *:::*::** :. ..*           :: *.. .   .    .*        *:: :**

LMD-3 239 GEIEIAKNEEVAFEAGSVDSNHSPKFGSDIALPAISEETKDSPCEEAKGDLSRNRSATLGDEEIQLRAR- 307
NCOA7 337 GEKIMTSDSRPIV-PLEKSTGHTPTKPSGSS------------VSEKLKKLDSSRETSHGSPTVTKLSKE 393
          **  ::.:..  .   . .:.*:*.  *. :             .*   .*. .*.:: *.  :   ::

LMD-3 307 -SRTSSQASSN-----DERPRSFSELDTPSEGKNIG-----------RFSPNAARRSFGKLGRTLSARAK 361
NCOA7 394 PSDTSSAFESTAKENFLGEDDDFVDLEELSSQTGGGMHKKDTLKECLSLDPEERKKAESQINNS--AVEM 461
           * ***  .*.       .  .* :*:  *. .. *            :.*:  ::: .:..:  *

LMD-3 362 SIQGTVTSGAEKVVGTA----VQGTKTV----AHGVVTHTKSAADTLQSGIEN----------------- 407
NCOA7 462 QVQS-----ALAFLGTENDVELKGALDLETCEKQDIMPEVDKQSGSPESRVENTLNIHEDLDKVKLIEYY 526
          .:*.     *  .:**     ::*:  :     :.:: .... :.: :* :**

LMD-3 407 ------GAKAAADVAGRVVDKGQSLMSESIN------------GVTEIFSVPDFEQ-PAKSPLAMKREQS 457
NCOA7 527 LTKNKEGPQVSENLQKTELSDGKSIEPGGIDITLSSSLSQAGDPITEGNKEPDKTWVKKGEPLPVKLNSS 596
                * :.: ::    :..*:*:   .*:             :**  . **       .** :* :.*

LMD-3 578 LAKLQDLRRQTAEARENSAKENHAGVFACATSS-D--EAPDLFAAVDEIMERNRIQSNDSTASQSPILPF 523
NCOA7 597 TE-A-NVIKEALDSSLESTLD-----NSCQGAQMDNKSEVQLWLLKRIQVPIEDILP--SKEEKSKTPPM 657
               :: ::: ::  :*: :      :*  :. *  .  :*:      :  : *    *. .:*   *:

LMD-3 524 YMAVRLTRNKKKKKSSTSSPSYDEDIAFG-NKLKREFWFAVPRNQADNIYHFLLQWSPDKYGLDTTTSTT 587
NCOA7 658 FLCIKVGKPMRKSFATHT---AAMVQQYGKRRKQPEYWFAVPRERVDHLYTFFVQWSPDVYGKDAK---- 720
          ::.::: :  :*. :: :         :* .: : *:******::.*::* *::***** ** *:.

LMD-3 588 EEPSSVMSNDGQDKGFIVLGSNADESLGGEKSTPLFGSSLLNREWELVTVSEMCRRLSLD----QELELP 658
NCOA7 720 ------------EQGFVVVEKEELNMI------DNFFSEPTTKSWEIITVEEAKRRKSTCSYYEDEDEEV 772
                      ::**:*: .:  : :        * *.  .:.**::**.*  ** *      :* *
                          TLDc
LMD-3 659 IPDGAATSQILDELMIRQVMDILPPRAEGYPWVNIYNSEKHGFSLATMYRKMAEFDEDLSPVLLIIRDTK 728
NCOA7 773 LPVLRPHSALLENMHIEQLARRLPARVQGYPWRLAYSTLEHGTSLKTLYRKSASLD---SPVLLVIKDMD 839
          :*     * :*::: *.*:   ** *.:****   *.: :** ** *:*** *.:*   *****:*:*
                                      W804C

LMD-3 729 EHVFGAVVSSAIRPNDHFFGTGDSCLLWRFTGEVPHTRELRQYNWTGDNQYFVNAAKDSLSIGAGSGRNG 798
NCOA7 840 NQIFGAYATHPFKFSDHYYGTGETFLY-TF------SPHFKVFKWSGENSYFINGDISSLELGGGGGRFG 902
          .:::*** .: :: .**::***:: *   *      : .:: ::*:*:*.**:*.  .**.:*.*.** *

LMD-3 799 LWLDADLNHGSSQKCETFDNEPLCGDDQDFIIQFIEAYGFRM 840
NCOA7 903 LWLDADLYHGRSNSCSTFNNDILS-KKEDFIVQDLEVWAFD* 942
          ******* ** *:.*.**:*: *. ..:***:* :*.:.*
```

S1 Fig. Sequence alignment highlighting conserved domains and the POI-associated residue.

Amino acid sequence alignments with the Clustal Omega program. The conserved LysM, GRAM, and TLDc domains are indicated by different colored backgrounds. Mutations associated with premature ovarian insufficiency (POI) are highlighted in red. The percentage of identical amino acids is 26.46%. Identical amino acids are marked with an asterisk (*), while strong and weak conservation are indicated with a colon (:) and a period (.), respectively.

S2 Fig

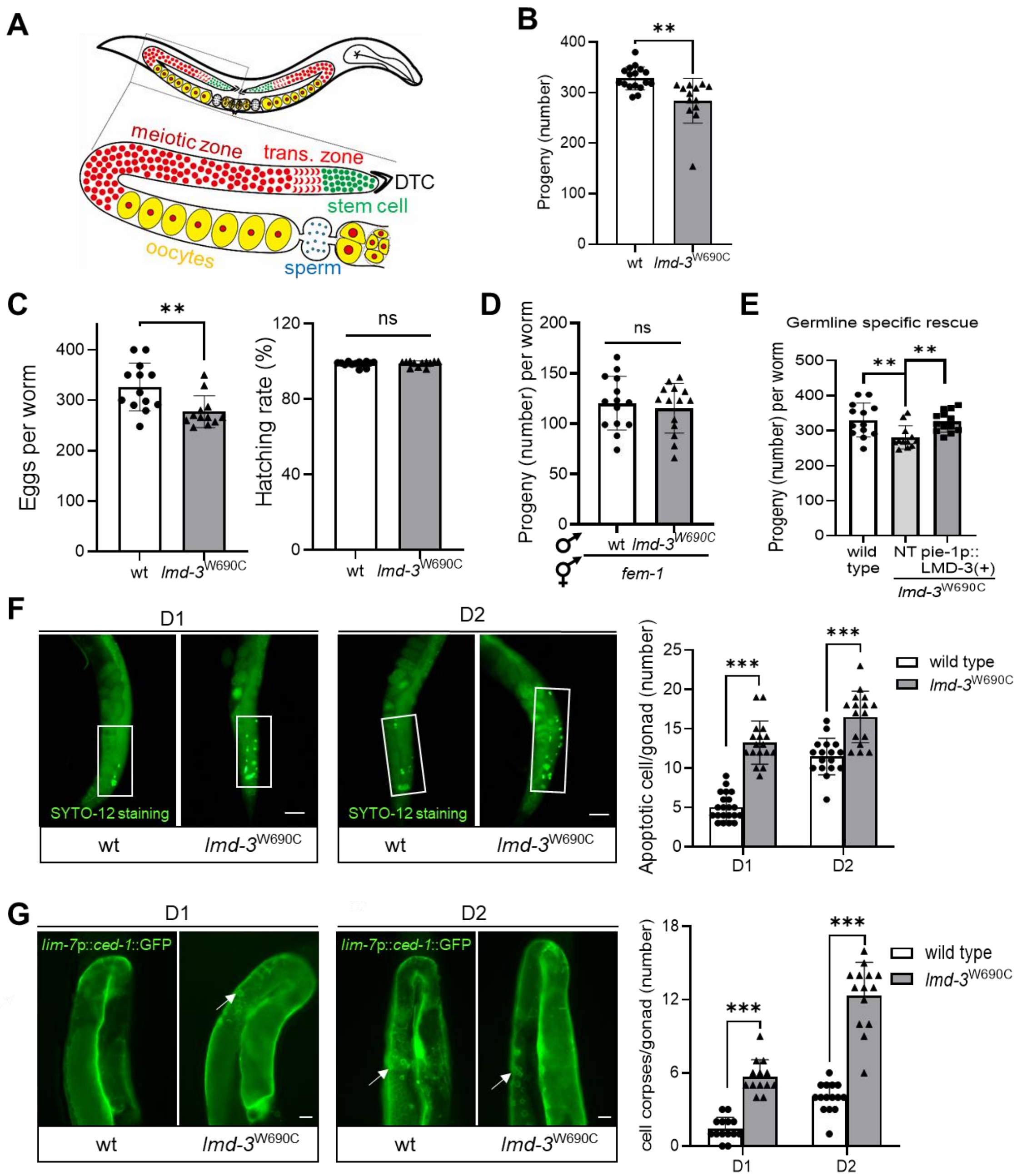

S2 Fig. The *lmd-3*^{W690C} mutation in *C. elegans* is associated with impaired fecundity and increased germline apoptosis.

(A) Spatial organization of the *C. elegans* germline stem cell and meiotic regions. The schematic diagram illustrates one of the two distinct gonad arms, showing the spatial organization of the stem cell region, transition zone, and the meiotic phase along the distal-proximal axis. The distal tip cell (DTC) is also depicted, highlighting its role in enclosing the distal arm. (B) Total brood sizes indicate the overall reproductive ability of wild-type and *lmd-3*^{W690C} mutants. Data are presented as mean ± SEM (n = 13 per group,

**$p < 0.01$, Cohen's $d = 1.322$). (C) Quantification of total eggs laid per worm (left) and hatching rate (right) for wild-type and *lmd-3*$^{\text{W690C}}$ mutants. The hatching rate was calculated as the percentage of viable progeny relative to the total number of eggs laid. Data are presented as mean ± SEM (n ≥ 12 per group, **$p < 0.01$ with Cohen's $d = 1.204$; ns, no significant differences with Cohen's $d = 0.074$). (D) Cross-fertilization assay evaluating sperm functionality. Total brood sizes were quantified for spermless *fem-1(hc17)* hermaphrodites mated with either wild-type or *lmd-3*$^{\text{W690C}}$ males at the restrictive 25 °C (n ≥ 14 for each group, ns, no significant differences, Cohen's $d = 0.195$). (E) Germline-specific rescue of the reproductive defect. Total progeny counts were assessed for wild-type, non-transgenic (NT) *lmd-3*$^{\text{W690C}}$ mutants, and *lmd-3*$^{\text{W690C}}$ mutant animals carrying a germline-specific expression array *pie-1*p::mCherry::LMD-3(+). NT, no transgene; Ex, extrachromosomal transgene. n ≥ 12 for each group, **$p < 0.01$, with effect sizes of Cohen's $d = 1.188$ between wild-type and NT mutants, and Cohen's $d = 1.439$ between NT mutants and *pie-1* rescue animals. (F, G) Independent replicates of germline apoptosis in wild-type and *lmd-3*$^{\text{W690C}}$ mutants with SYTO12 staining (F) and (G) a *lim-7*p::*ced-1*::GFP reporter (n ≥ 13 per group; unpaired t-tests, ***$p < 0.001$), with effect sizes for (F) being Cohen's $d = 3.955$ at D1 and Cohen's $d = 1.932$ at D2, and for (G) being Cohen's $d = 3.646$ at D1 and Cohen's $d = 4.046$ at D2. White arrowheads indicate apoptotic cell corpses. The data show a significant increase in apoptosis in the mutants. D1, Day 1 of adulthood; D2, Day 2 of adulthood. Scale bars, 50 µm.

S3 Fig

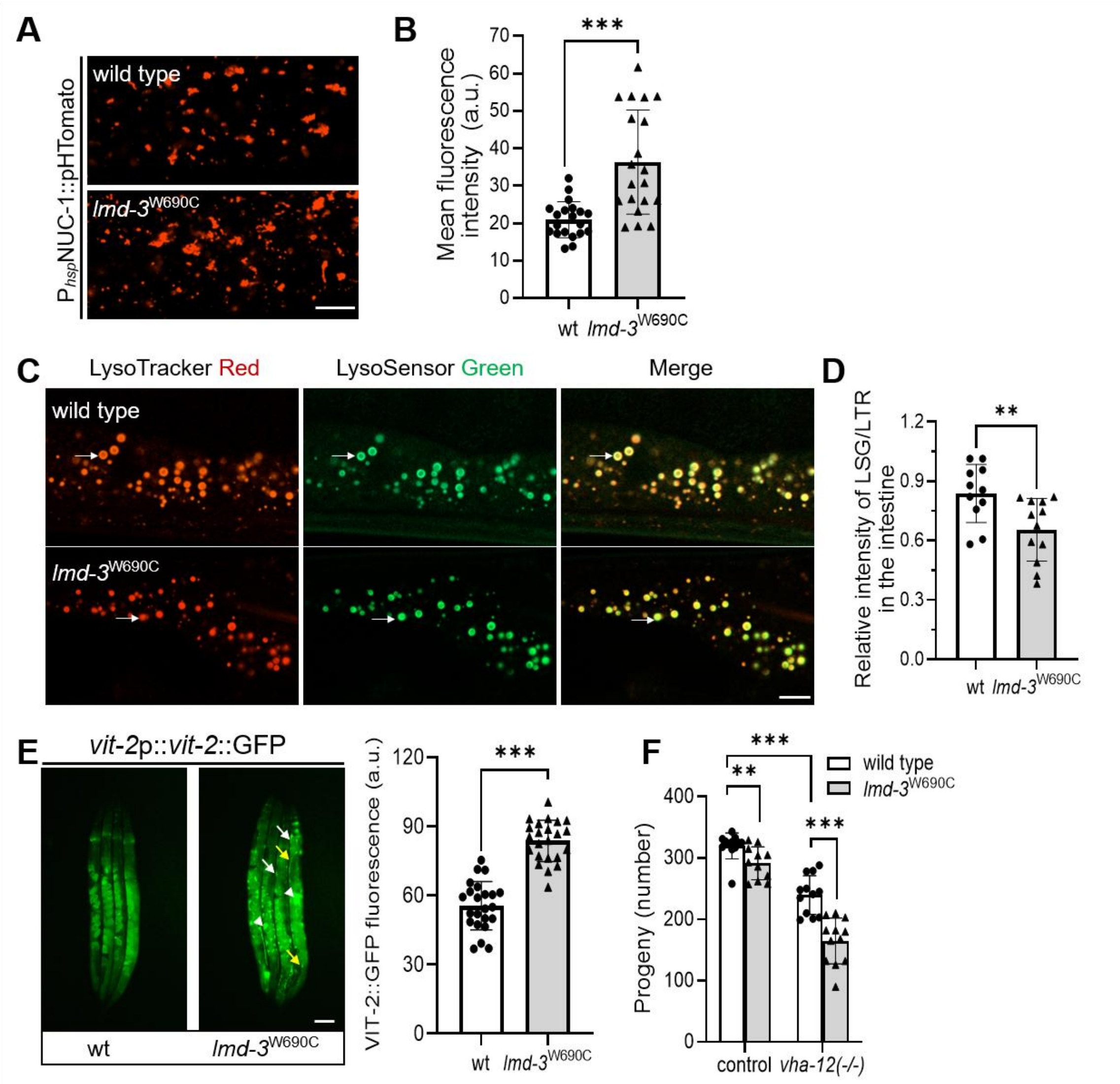

S3 Fig. The W690C mutation correlates with impaired protein degradation and lysosomal function.

(A, B) Independent replicates of fluorescence images (A) and mean fluorescence intensity quantification (B) of NUC-1::pHTomato expression in the hypodermis driven by a heat-shock (hs) promoter (n = 20 per group, ****p* < 0.001, Cohen's *d* = 1.480). a.u., arbitrary units. Scale bar, 10 μm. (C, D) Independent replicates of confocal fluorescence images (C) and quantification (D) of intestines co-stained with Lysosome-Specific Green (LSG DND-189) and Lysosome-Targeting Red (LTR DND-99) in wild type and *lmd-3*^{W690C} animals at the D1 adult stage. White arrowheads indicate vesicular lysosomes stained by both dyes. Relative intensity of LSG/LTR were quantified (n ≥ 11 per group, ***p* < 0.01, Cohen's *d* = 1.202). Scale bars, 10 μm. (E) Representative fluorescence images and quantification of VIT-2::GFP accumulation in wild type and *lmd-3*^{W690C} animals at D1 adult stage (n = 23 per group, unpaired t-tests: ****p* < 0.001, Cohen's *d* = 3.035). Aggregates in different tissues: white arrowheads denote germline, white arrows denote pseudocoelom,

yellow arrows denote intestines. a.u., arbitrary units. Scale bars, 50 μm. (F) Total brood sizes indicate the effect of *vha-12(ok821)* mutants on reproductive capacity in wild type and *lmd-3*^{W690C} animals (n ≥ 11 per group, ***p* < 0.01, ****p* < 0.001, with effect sizes of Cohen's *d* = 1.192 for control, Cohen's *d* = 2.168 for *vha-12* mutants, and Cohen's *d* = 2.991 between control and *vha-12* groups).

S4 Fig

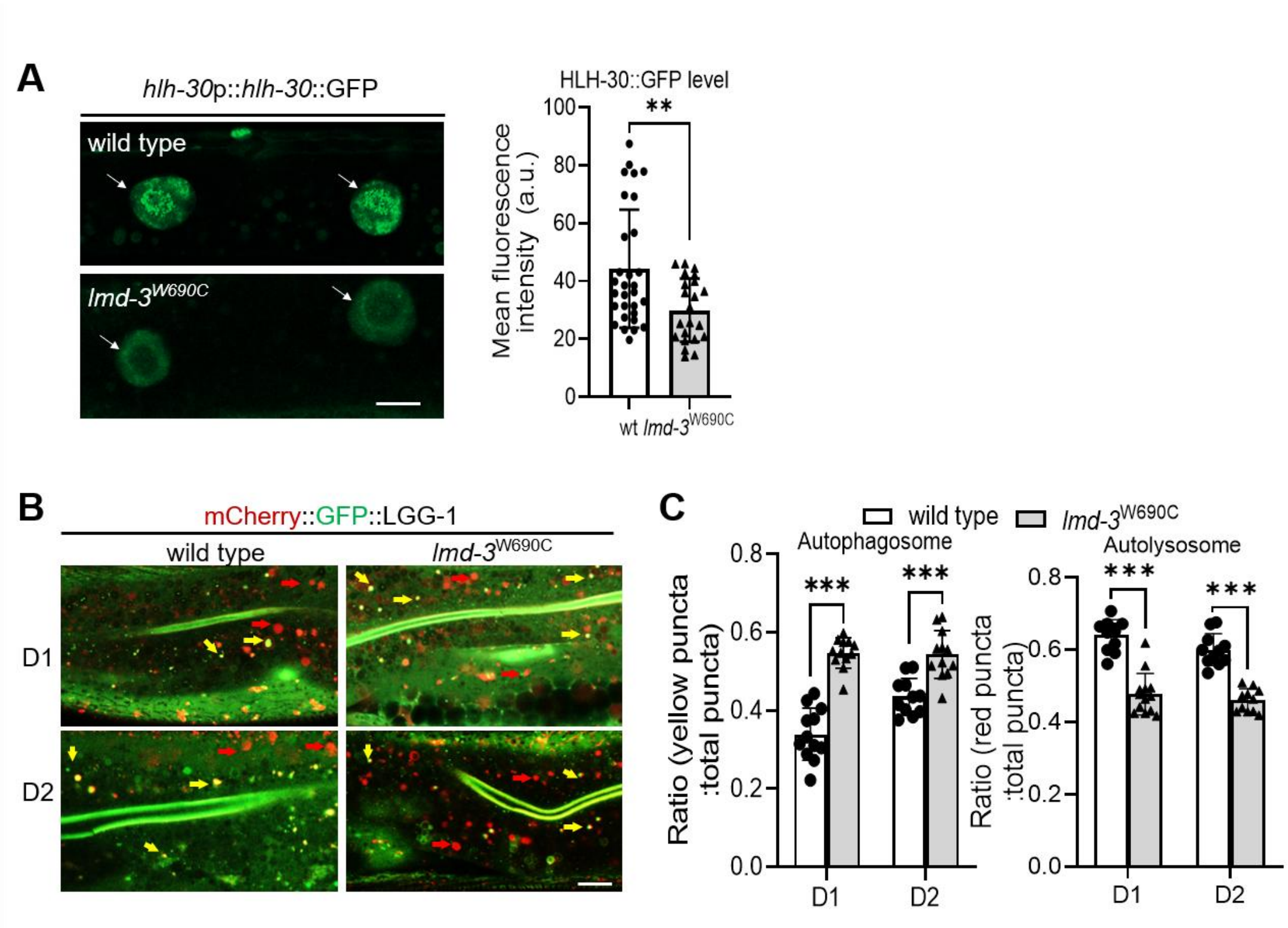

S4 Fig. *lmd-3*^{W690C} mutation is associated with impaired autophagic transcription and autophagic flux.

(A) Confocal images and mean fluorescence intensity quantification of the nuclear intensity of HLH-30::GFP in D1-stage with wild type and *lmd-3*^{W690C} mutants. White arrows indicate the nuclear localization of HLH-30::GFP (n ≥ 22 per group, **$p < 0.01$, Cohen's $d = 0.840$). Scale bar, 10 μm. a.u., arbitrary units. (B, C) Independent replicates of confocal images (B) and quantification (C) of the mCherry::GFP::LGG-1 reporter in wild type and *lmd-3*^{W690C} animals at D1 and D2 adult stages. The ratio of autophagosomes (yellow puncta, yellow arrow) to total puncta and autolysosomes (red puncta, red arrow) to total puncta was quantified (n ≥ 11 per group, ***$p < 0.001$, with effect sizes for autophagosomes being Cohen's $d = 3.824$ at D1 and Cohen's $d = 1.968$ at D2, and for autolysosomes being Cohen's $d = 3.174$ at D1 and Cohen's $d = 3.626$ at D2). Scale bar, 10 μm.

S5 Fig

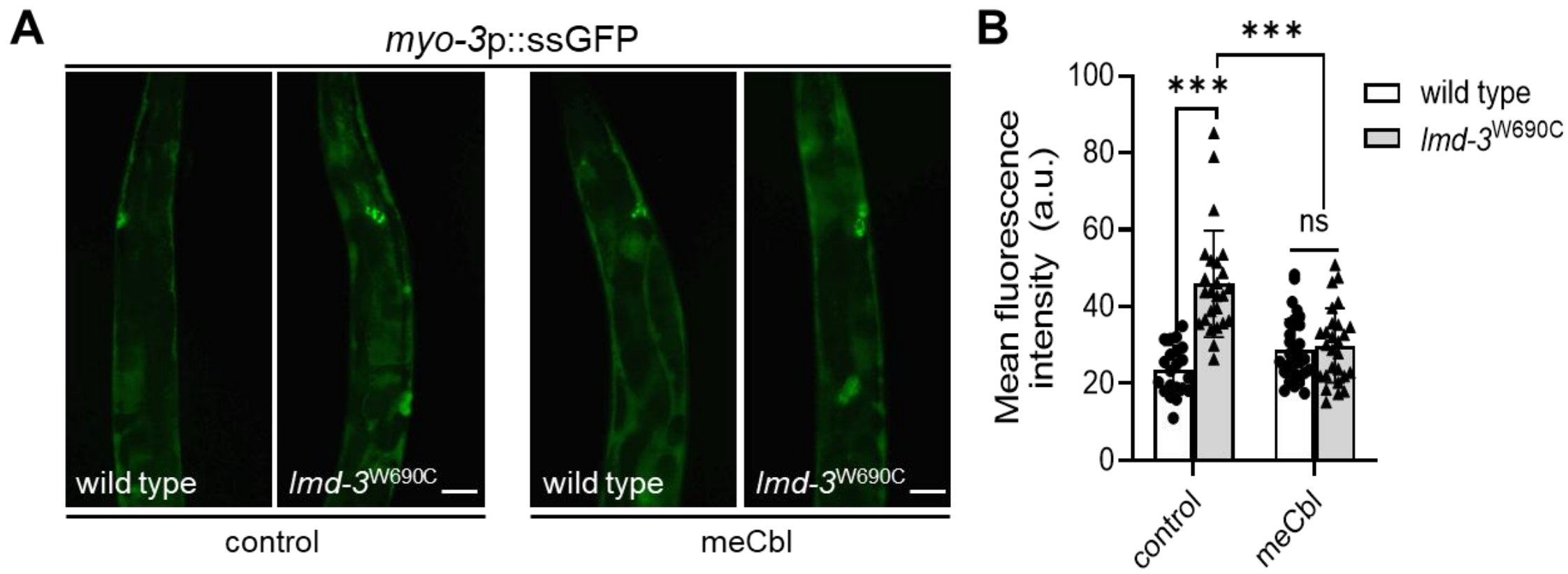

S5 Fig. Mecobalamin rescues secreted protein accumulation defects in *lmd-3*^{W690C} mutants.

(A, B) Representative fluorescence images (A) and mean fluorescence intensity quantification (B) of secreted protein accumulation (*myo-3*p::ssGFP) in the coelomocytes of wild type and *lmd-3*^{W690C} animals with mecobalamin (meCbl) supplementation at the D1 stages (mean ± SEM, n ≥ 21 per group, unpaired t-tests: ***p < 0.001, ns, no significant differences, with effect sizes of Cohen's d = 2.017 between wild type and mutant under control, Cohen's d = 0.135 under meCbl, and Cohen's d = 1.355 between control and meCbl-treated *lmd-3*^{W690C} mutants). Scale bars, 50 µm. a.u., arbitrary units.

S6 Fig

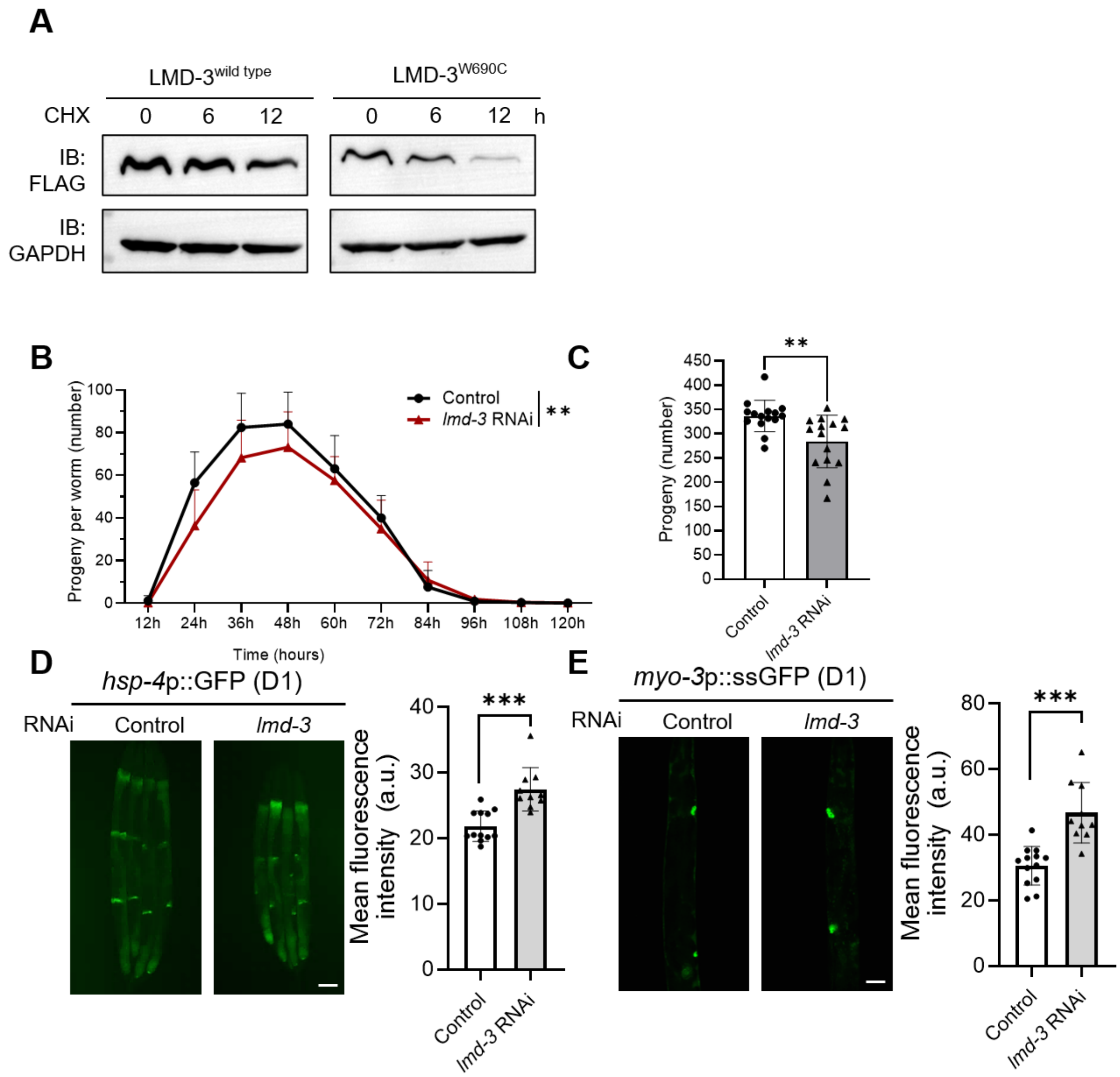

S6 Fig. The W690C mutation reduces *lmd-3* transcript levels and correlates with compromised protein stability.
(A) Independent replicates of Western blot images show the turnover of 1xFLAG-tagged LMD-3 W690C mutation in HEK-293T cells treated with cycloheximide (CHX) for 0, 6, and 12 h. (B) Progeny production curves show the reproductive output of control and *lmd-3* RNAi hermaphrodites (n = 15 per group, $^{**}p < 0.01$). (C) Total brood sizes indicate the overall reproductive ability of control and *lmd-3* RNAi hermaphrodites. Data are presented as mean ± SEM (n = 15 per group, $^{**}p < 0.01$, Cohen's $d = 1.175$). (D) Exemplar fluorescence images and mean fluorescence intensity quantification of the UPR^{ER} reporter *hsp-4*p::GFP animals with control and *lmd-3* RNAi at D1 stages (n ≥ 10 for each group, unpaired t-tests, $^{***}p < 0.001$, Cohen's $d = 2.004$). Scale bars represent 100 μm. a.u., arbitrary units. (E) Exemplar fluorescence images and mean fluorescence intensity quantification of secreted protein (*myo-3*p::ssGFP) accumulation in the coelomocyte of

animals with control and *lmd-3* RNAi at D1 stages ($n \geq 10$ per group, unpaired t-tests: ***$p < 0.001$, Cohen's $d = 2.154$). Scale bars, 50 μm. a.u., arbitrary units.

S2 Table

S2 Table. Summary of examined reporters and associated germline defects in *lmd-3*^{W690C} mutants.

	*hsp-16.2*p::GFP (UPR^{cyto}) intensity		*hsp-4p::GFP* (UPR^{ER}) intensity		Progeny number	Germline stem cells number	Proliferative zone nuclei number	Meiotic prophase nuclei number
wild type	L4	32.96±3.89	L4	29.98±3.75	332.59±24.00	455.59±13.18	182.29±12.37	301.43±33.32
	D1	34.26±3.05	D1	33.60±5.77				
	D2	34.50±2.63	D2	37.83±6.23				
lmd-3^{W690C}	L4	31.63±3.31	L4	29.05±5.13	225.76±73.36	418.60±14.26	137.14±15.54	266.93±30.23
	D1	33.99±2.18	D1	37.25±6.42				
	D2	38.93±4.91	D2	44.73±4.62				

S4 Table

S4 Table. Machine learning-based prediction of LMD-3^{W690C} stability

Tool Name	ΔΔG (Kcal/mol)	Outcome	Web server
mCSM[a]	-1.458	Destabilizing	https://biosig.lab.uq.edu.au/duet/
SDM[a]	-1.64	Destabilizing	https://biosig.lab.uq.edu.au/duet/
DUET[a]	-1.286	Destabilizing	https://biosig.lab.uq.edu.au/duet/
INPS (sequence)[a]	-1.64	Destabilizing	http://inpsmd.biocomp.unibo.it.
INPS (structure)[a]	-2.15	Destabilizing	http://inpsmd.biocomp.unibo.it.
I-Mutant (sequence)[b]	-1.63	Destabilizing	https://folding.biofold.org/i-mutant/i-mutant2.0.html

Six machine learning-based methods were used to predict the change in free energy (ΔΔG) upon W690C mutation. [a], A negative ΔΔG indicates structural destabilization; [b], $\Delta\Delta G < -0.5$ indicates the decrease of stability.

S1 Video. Structural conservation of the TLDc domains. This movie presents a 360-degree rotation of the aligned TLDc domains from human NCOA7 (pink) and *C. elegans* LMD-3 (light blue). The movie highlights the remarkable structural conservation, with key conserved tryptophan residues (human W804 and *C. elegans* W690) highlighted by black arrows and colored bright green and yellow, respectively. (Data provided in a separate .mp4 format file)

S1 Table. *C. elegans* strains used in this study. (Data provided in a separate Excel spreadsheet)

S3 Table. Primers and oligos used in this study. (Data provided in a separate Excel spreadsheet)

S1 Data. Comprehensive record of experimental raw data. (Data provided in a separate Excel spreadsheet)

S2 Data. Uncropped scans of western blot membranes. (Images provided in a separate Word document)
