# Supplementary material for: A premature ovarian insufficiency-associated LMD-3 point mutation impairs protein stability and causes autophagic-lysosomal dysfunction in Caenorhabditis elegans
Source: PLoS One. 2026 Oct 27;21(10):e0361215. doi: 10.1371/journal.pone.0361215 (PMC13712518; doi:10.1371/journal.pone.0361215)
Supplement: Supplementary file 2 [file pone.0361215.s008.pdf]

Response to Reviews:
We are grateful to the reviewers for their constructive and insightful feedback on our manuscript. We are encouraged that they recognized the value of our *C. elegans* POI model. In response to their suggestions, we have increased RT-qPCR biological replicates, optimized image quality, and included effect size measurements. More importantly, we have restructured the Results and Discussion sections, systematically moderated our translational and vitamin B12-related claims, framed the proposed pathway as a working mechanistic model, and resolved all technical formatting and raw data requirements. A detailed, point-by-point response to all comments is provided below.

Reviewer #1: Recommendation: Major Revision
Major Comments
1. Although the study successfully establishes a C. elegans model carrying the POI-associated mutation, the translational relevance to human disease remains insufficiently supported. The manuscript would benefit from additional experimental evidence or a more cautious interpretation of the findings regarding human POI.

We sincerely thank the reviewer for this insightful and critical comment. We completely agree that while *C. elegans* is a powerful in vivo platform for dissecting conserved molecular mechanisms, caution must be exercised when extrapolating findings from nematodes to human clinical pathologies such as premature ovarian insufficiency (POI).
In response to this constructive suggestion, we have thoroughly reviewed the manuscript and made the following comprehensive revisions to ensure a more cautious and rigorous interpretation:
a. Systematic softening of translational claims:
We have removed or modified definitive statements regarding vitamin B12/mecobalamin as a direct "translatable therapeutic intervention" for human patients. Throughout the revised Abstract, Introduction, and Discussion sections, we now explicitly frame our findings as providing mechanistic insights into evolutionarily conserved pathways and potential metabolic leads rather than direct clinical treatments.
b. Explicit acknowledgment of model limitations in the discussion:
We have expanded the Discussion section to further emphasize the biological and physiological boundaries of the *C. elegans* model when applied to human ovarian biology. Specifically, we now clearly state that while nematodes share core proteostatic and lysosomal regulatory machineries with humans, organismal complexity, ovarian architecture, and systemic metabolism differ significantly. Therefore, the therapeutic potential of vitamin B12 observed in our model serves as a conceptual and metabolic framework that warrants rigorous future validation in mammalian models and clinical studies.
We believe these modifications appropriately balance our mechanistic discoveries with the necessary caution regarding their clinical translation, thereby strengthening the overall scientific rigor of the manuscript.

2. The authors report that the W690C mutation reduces both LMD-3 protein stability and transcript levels. However, the mechanism underlying the reduction in mRNA expression is not investigated. Additional experiments or a more detailed discussion addressing this observation would strengthen the mechanistic conclusions.

We thank the reviewer for highlighting this important point. To ensure the robustness of this observation, we first performed additional independent RT-qPCR experiments, increasing our biological replicates from n=3 to n=4. These new data consistently confirm the significant reduction in *lmd-3* transcript levels in the W690C mutant, as updated in new Fig 7B (see below Fig for reviewer).

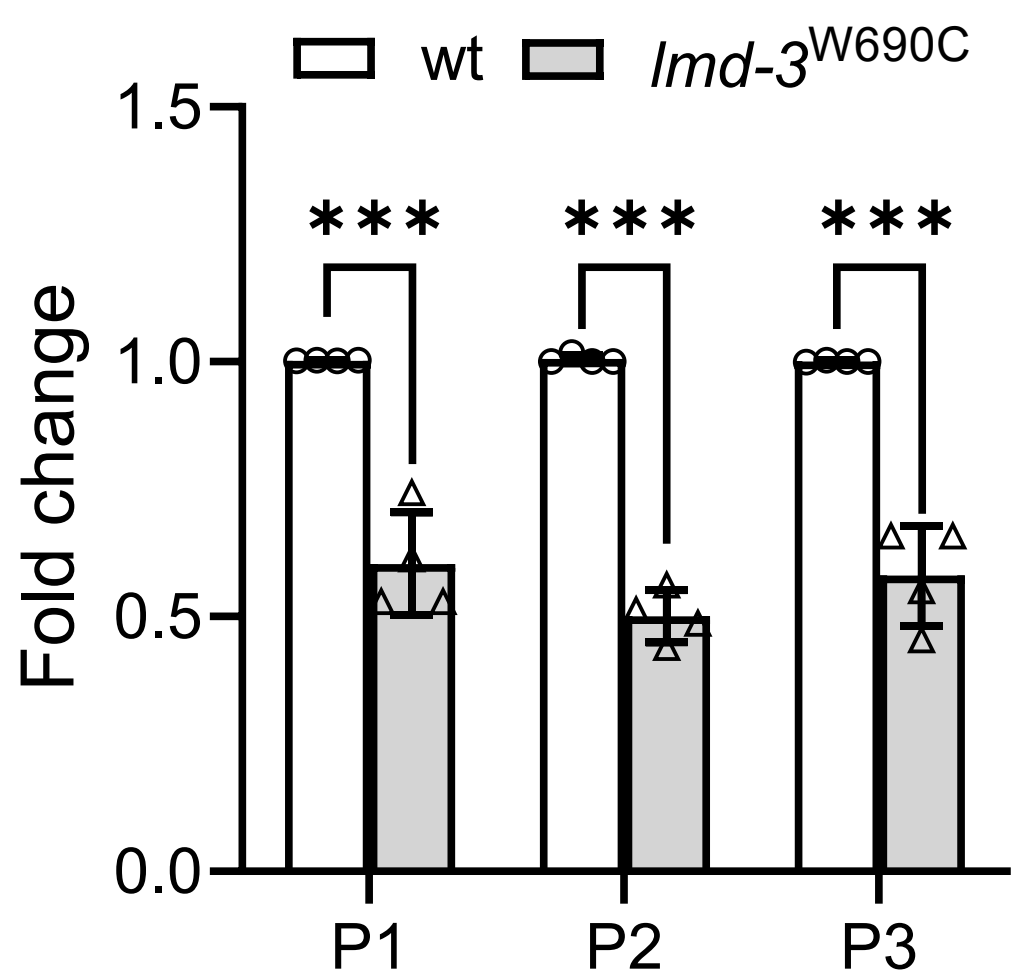

We agree with the reviewer that discussing the potential mechanism driving this mRNA reduction is critical. As suggested, we have expanded our Discussion to address this observation. Specifically, we propose that the single-nucleotide missense mutation may alter the local secondary structure or thermodynamic stability of the *lmd-3* transcript, thereby triggering cellular mRNA surveillance and degradation pathways, as described in "*Cell.* 2009 Feb 20;136(4):763-76. doi: 10.1016/j.cell.2009.01.019." Furthermore, we contextualize the difference between our findings and the stable transcript levels reported in human NCOA7(W804C) models as reflecting widely documented species-specific variations in mRNA regulation.
These revisions have been incorporated into the Discussion section (Paragraph 2) of the revised manuscript. We believe this updated theoretical framework logically addresses the reviewer's concern and provides a clear direction for future studies without overextending our current definitive conclusions.

3. The conclusion that lysosomal dysfunction induces a functional vitamin B12 deficiency is based primarily on indirect evidence. Direct biochemical measurements of vitamin B12 levels or related metabolic markers would provide stronger support for this important claim.

We appreciate the reviewer's rigorous perspective and fully agree that direct biochemical quantification, such as mass spectrometry for intracellular B12, propionate, or methylmalonic acid, is the ultimate gold standard for measuring absolute metabolite concentrations.
However, in *C. elegans* research, direct measurement of organismal B12 can be technically confounded by the *E. coli* diet (OP50) present in the gut. To circumvent this, the field widely relies on the *acdh-1*p::GFP reporter as a highly sensitive and specific *in vivo* biosensor for functional B12 deficiency, as the reference we originally cited in "*Cell*. 2013 Mar;153(1):240-52. doi: 10.1016/j.cell.2013.02.049." and "*Elife*. 2016 Jul 6:5:e17670. doi: 10.7554/eLife.17670." Mechanistically, when intracellular B12 levels drop, the canonical propionate breakdown pathway is blocked, leading to the accumulation of toxic propionate. This accumulation obligatorily triggers the robust transcriptional activation of the compensatory propionate shunt pathway, heavily marked by *acdh-1* induction. Therefore, while indirect, the strong induction of the *acdh-1* reporter in our mutants robustly reflects a state where the cells are functionally starved of B12.
Nevertheless, we completely accept the reviewer's critique that our current evidence remains technically indirect. To ensure scientific rigor and avoid overstating our findings, we have made two specific revisions to the manuscript:
a. We have softened the language in the Results section, changing "confirming" to "strongly indicating a state of functional vitamin B12 deficiency."
b. We have explicitly added the reliance on this indirect in vivo biosensor as a formal limitation in the Discussion section, noting that future direct biochemical measurements will be valuable to further substantiate these metabolic shifts.
We believe these modifications accurately contextualize our findings and address your valid concern.

4. The proposed mechanistic pathway linking LMD-3, V-ATPase dysfunction, autophagy impairment, and reproductive decline would be strengthened by additional genetic interaction (epistasis) experiments or complementary functional analyses.

We thank the reviewer for this insightful suggestion. We completely agree that mapping the genetic interaction network is crucial. However, this foundational genetic framework, which establishes that LMD-3 acts cooperatively with V-ATPase and other genetic interactors to control lysosomal and reproductive homeostasis, was systematically mapped and validated using null alleles in our previous work "*Sci Adv*. 2025 Nov;11(48):eadz3192.doi: 10.1126/sciadv.adz3192." Building upon that established broad genetic network, the specific rationale and core novelty of the present study lie in addressing a critical clinical translational gap to determine how a single, patient-derived missense mutation (W690C, orthologous to human NCOA7 W804C) specifically impacts molecular pathology. Unlike a complete gene deletion, our current data demonstrate that the primary pathogenic driver of this clinical variant is protein structural destabilization and rapid turnover (Fig. 7), rather than the formation of a novel macro-genetic network. Therefore, performing a secondary, exhaustive epistasis matrix specifically for this

point mutation would be largely redundant with our established framework. Instead, to avoid overclaiming and to respect the reviewer's perspective, we have explicitly contextualized this interconnected cascade as a proposed mechanistic model in the Discussion. This revision keeps the current study tightly focused on its true novelty, which is the variant-specific biochemical collapse and its metabolic rescue by vitamin B12.

5. Several conclusions, particularly those suggesting that vitamin B12 represents a "translatable therapeutic intervention," appear stronger than the presented data support. Since all experiments were performed in C. elegans, these statements should be moderated.

We sincerely thank the reviewer for this insightful and prudent feedback. We completely agree that describing vitamin B12 supplementation as a direct "translatable therapeutic intervention" overstates the current scope of our findings, given that all data were generated exclusively in *C. elegans*.
To address this concern rigorously, we have systematically moderated our conclusions across the entire manuscript:
a. Abstract:
We have replaced definitive therapeutic claims with a more cautious interpretation, now stating that our findings suggest vitamin B12 supplementation as a "promising metabolic intervention worth further translational exploration."
b. Discussion:
We have explicitly tempered our closing statements. Instead of framing vitamin B12 as a definitive clinical treatment, we now conclude that our work highlights vitamin B12 as a "valuable conceptual framework for understanding and potentially managing early-onset reproductive decline, pending future translational studies."
c. Consistency Check:
We have thoroughly screened the manuscript to eliminate any remaining phrasing that implies direct clinical efficacy, ensuring that the therapeutic potential of vitamin B12 is strictly and accurately contextualized as a metabolic lead for future mammalian and clinical validation.
We believe these modifications properly calibrate the tone of our conclusions to reflect the experimental boundaries of our nematode model.

Minor Comments:
1. The quality of several fluorescence microscopy images should be improved to facilitate data interpretation.

We sincerely thank the reviewer for this constructive suggestion. To facilitate clearer data interpretation and enhance the overall visual quality, we have re-evaluated, re-captured, and optimized the fluorescence microscopy images across the manuscript. Specifically, we have improved the resolution, contrast, and signal-to-noise ratio for the following key panels:
a. Figure 4 H-I:
We have optimized the imaging parameters for *nmy-2*p::*nmy-2*::GFP and *unc-*

*54*p::Q40::YFP, making the protein aggregation phenotypes in the *lmd-3* mutant background much sharper and clearer for visualization.

b. Figure S3F:

The *vit-2*p::*vit-2*::GFP images have been re-captured with enhanced contrast. The distinct tissue-specific aggregation patterns, highlighted by white arrowheads (germline), white arrows (pseudocoelom), and yellow arrows (intestines), are now much more distinct and easier to interpret.

c. Figure 5C:

We have improved the clarity of the *sqst-1*p::*sqst-1*::GFP images, ensuring that the fluorescent protein aggregates in the head neurons are sharp and well-defined.

We believe these upgraded, high-quality images substantially improve the visual presentation and readability of our data.

2. Some quantitative analyses, particularly RT-qPCR experiments with only three biological replicates, would benefit from additional replication to improve statistical robustness.

We sincerely thank the reviewer for this constructive suggestion. To enhance the statistical robustness of our transcriptional data, we have performed additional independent experiments, successfully increasing our biological replicates from n=3 to n=4 for all RT-qPCR analyses throughout the study (see below Fig for reviewer).

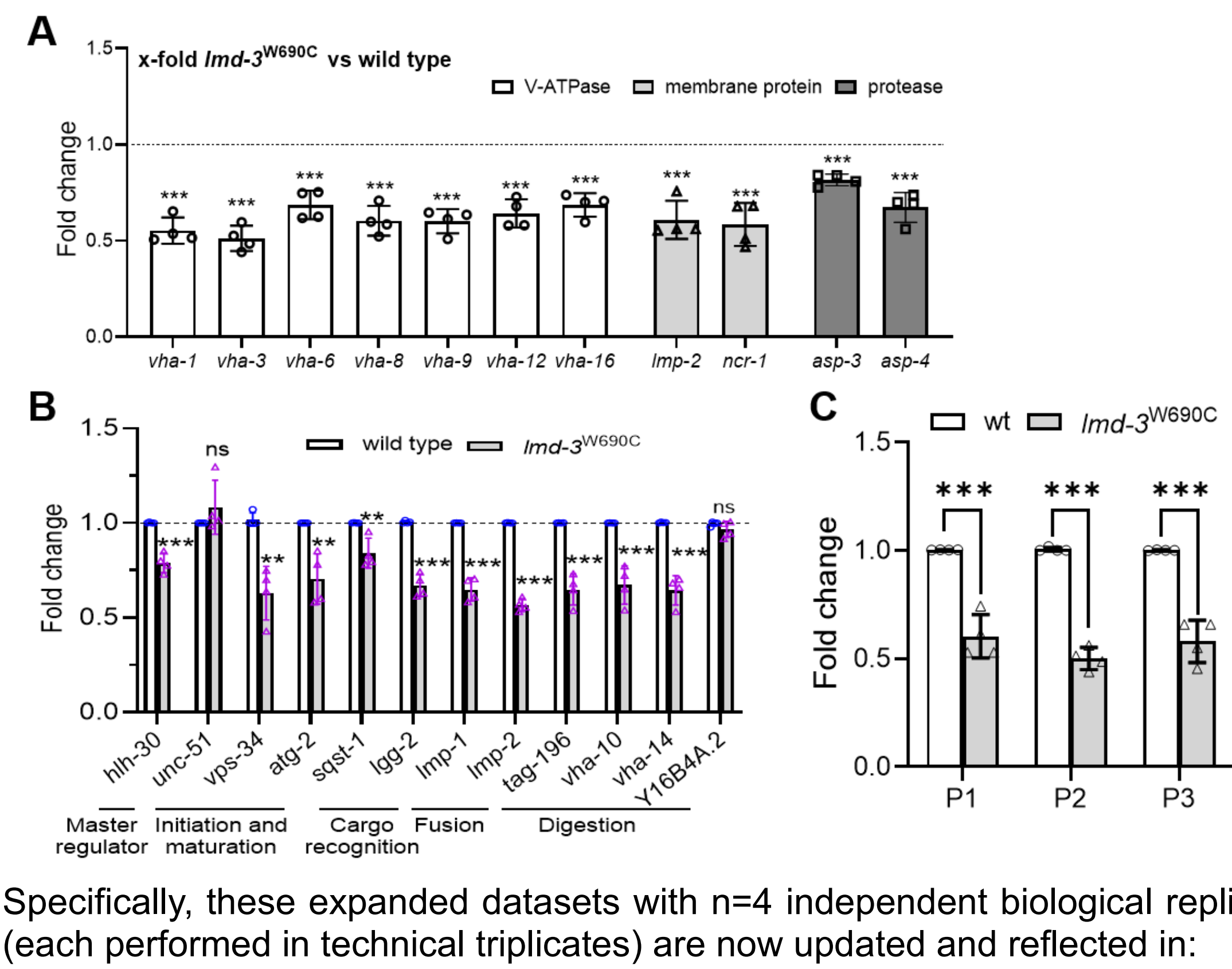

Specifically, these expanded datasets with n=4 independent biological replicates (each performed in technical triplicates) are now updated and reflected in:

a. Figure 4A: Transcriptional analysis of lysosome-related genes.

b. Figure 5B: Transcriptional analysis of key autophagy genes.
c. Figure 7B: Transcriptional analysis of endogenous *lmd-3* mRNA levels.
These additional replicates consistently validate our original findings, significantly strengthening the statistical reliability and rigor of our quantitative conclusions.

3. The Discussion should further emphasize the limitations of the C. elegans model when extrapolating findings to human ovarian biology.

We sincerely thank the reviewer for this constructive advice. In the revised Discussion section, we have further expanded our evaluation of the biological boundaries and limitations of the *C. elegans* model when translating findings to human ovarian aging. Specifically, we have explicitly incorporated discussion on:
a. Anatomical and structural differences:
The distinct architecture of the nematode germline (characterized by continuous germline proliferation and a syncytial organization) versus the finite pool of primordial follicles in human ovaries.
b. Drivers of reproductive decline:
While mammalian ovarian aging is primarily driven by oocyte exhaustion and atresia, *C. elegans* reproductive decline involves a complex interplay of both oocyte quality and physiological aging factors.
c. Systemic versus local metabolic control:
The systemic nature of our current phenotypic assays compared to the cell-type-specific microenvironment of human granulosa and oocyte interactions.
d. Indirect metabolic assessment:
The reliance on the *acdh-1* in vivo biosensor to infer vitamin B12 deficiency, highlighting the need for future direct biochemical quantification.
We believe these additions provide a more rigorous, balanced, and scientifically cautious context for interpreting our nematode-based findings in relation to human ovarian biology.

4. Several sentences in the Introduction and Discussion are overly long and could be revised for improved readability.

We are highly appreciative of the reviewer’s constructive feedback regarding the readability of the manuscript. We fully agree that clarity and flow are paramount, and that some of our previous sentences were overly complex.
To address this, we have conducted a comprehensive review of the entire manuscript, with a specific focus on the Introduction and Discussion sections. We have systematically broken down lengthy, multi-clause sentences into shorter, more direct statements to improve narrative flow and eliminate run-on phrasing.
As examples of these structural improvements, we highlight the following specific revisions:
a. Example 1 (Introduction):
Original: "In women, ovarian function diminishes prematurely compared to other somatic systems [3,4], a process whose acceleration underlies premature ovarian insufficiency (POI), an increasingly global health concern [1,5]."

Revised: "In women, ovarian function diminishes prematurely compared to other somatic systems [3,4]. The acceleration of this process underlies premature ovarian insufficiency (POI), an increasingly global health concern [1,5]."
b. Example 2 (Discussion):
Original: "Building upon our previous work characterizing LMD-3 as a critical regulator of reproductive capacity in *C. elegans* [24], this study leverages a novel POI-associated W690C knock-in model to dissect the pathophysiological mechanisms of fertility decline at a single-amino-acid resolution."
Revised: "Our previous work established LMD-3 as a critical regulator of reproductive capacity in *C. elegans* [24]. Building upon this foundation, the current study leverages a novel POI-associated W690C knock-in model. This approach allows us to dissect the pathophysiological mechanisms of fertility decline at single-amino-acid resolution."
c. Example 3 (Discussion):
Original: "By defining this elegant link between molecular stability and reproductive health, we not only validate our *C. elegans* model as a platform for mechanistic discovery but also highlight vitamin B12 as a valuable conceptual framework for understanding..."
Revised: "By defining this elegant link between molecular stability and reproductive health, we validate our *C. elegans* model as a platform for mechanistic discovery. Furthermore, we highlight vitamin B12 as a valuable conceptual framework for understanding..."
We believe these stylistic revisions, along with numerous similar adjustments throughout the text, have substantially enhanced the readability and impact of the manuscript.

5. Reporting effect sizes in addition to p-values would improve the statistical presentation of the results.

We thank the reviewer for this constructive suggestion. We fully agree that reporting effect sizes alongside *p*-values offers a more rigorous and comprehensive evaluation of the magnitude of experimental effects.
To address this, we have systematically calculated the effect sizes (Cohen's *d*) for all pairwise comparisons across the manuscript. Given the extensive dataset, such as comprehensive qRT-PCR panels evaluating multiple lysosomal and autophagic genes, we have structured our presentation as follows to ensure clarity and readability:
a. Supplementary data integration:
For large-scale analyses and multi-group quantitative comparisons (**Fig 4A, Fig 5B, D, E, and Fig 6B, D, E**), the complete set of individual Cohen's *d* values has been compiled and presented in **S1 Data**.
b. Figure legend updates:
For primary phenotypic assays and key functional quantifications, such as brood sizes, germline apoptosis, and reporter intensities, the specific Cohen's *d* values have been directly incorporated into the respective figure legends.
c. Methodology update:

We have updated the Statistical analysis section in the Materials and methods to explicitly state: "To evaluate the magnitude of differences between groups, effect sizes were calculated using Cohen's *d* where appropriate."
We believe these comprehensive statistical additions significantly strengthen the quantitative rigor and transparency of our findings.

Overall Evaluation
This manuscript addresses an interesting and biologically relevant question using a comprehensive experimental approach. The data are generally of good quality, and the findings provide valuable insights into the role of LMD-3 in lysosomal function and reproductive aging. However, several mechanistic claims require stronger experimental support, and some conclusions should be presented more cautiously. Therefore, I recommend Major Revision before the manuscript can be considered for publication.

We sincerely thank the reviewer for this encouraging and positive overall assessment of our work. We deeply appreciate your recognition of the study's biological relevance, comprehensive approach, and data quality. As detailed in our point-by-point responses above, we have taken your constructive critiques to heart by significantly moderating our translational conclusions, framing the mechanistic pathways more cautiously, and bolstering the statistical and experimental rigor of our datasets. We believe these revisions have fundamentally strengthened the manuscript, and we are truly grateful for your time and expertise in helping us improve this study.

Reviewer #2:
This manuscript addresses a relevant topic—the impact of LMD-3 point mutation on premature ovarian insufficiency in C. elegans.
1. The introduction is well structured and allows a comprehensive overview of the recent advances related to the topic. However, authors described the findings of the current study at the end of the introduction section. These data are not relevant to the introduction; they should be removed "Our data show that this point mutation significantly reduces early reproductive capacity…..a potential therapeutic intervention".

We fully agree with the reviewer's professional insight regarding the standard structure of a scientific Introduction. Presenting detailed findings and conclusions at this stage prematurely disclosed the results and disrupted the narrative flow.
In response to this valuable feedback, we have completely removed the sentences summarizing our experimental data (from "Our data show that this point mutation…" to "…identifying a potential therapeutic intervention") from the Introduction. We have now strictly reserved data interpretation for the Results and Discussion sections. Furthermore, we have utilized this space to clearly formulate the specific aims of the current study, ensuring the Introduction appropriately sets the stage and rationale for our investigation without preempting the findings.

2. Authors should clearly formulate the aim of the study at the end of the introduction section.

We thank the reviewer for this excellent suggestion, which perfectly complements the previous point. We have completely rewritten the final paragraph of the Introduction to explicitly define the scientific aims and scope of our study, replacing the previous summary of results.

The revised concluding paragraph now clearly formulates our objectives as follows: "In this study, we modeled the clinically relevant human mutation by engineering the orthologous W690C substitution into the *C. elegans lmd-3* locus to probe its biochemical and pathogenic consequences. Specifically, we sought to determine how this single-amino-acid substitution impacts LMD-3 protein stability, systemic autophagic-lysosomal function, and reproductive capacity. Furthermore, we investigated whether metabolic supplementation could mitigate these mutational defects, thereby providing new insights into the cellular mechanisms underlying early-onset reproductive decline."

3. Some methodological points are stated in the results section (example: Employing two complementary approaches—the nucleic acid dye SYTO12 and a lim-7p::ced-1::GFP reporter [35,36], we observed….These data are to remove as they are related to the methods section.

We sincerely apologize for inappropriately including methodological details in the Results section. We fully agree with the reviewer that such descriptions disrupt the flow of the results and belong exclusively in the Materials and Methods section.

In the revised manuscript, we have strictly removed the specific sentence highlighted by the reviewer. It now reads: "We observed a significant elevation in apoptotic cell corpses within the germlines of *lmd-3*^{W690C} mutants at Day 1 and Day 2 of adulthood (Figs 2F, 2G; S2F and S2G Figs)."

Furthermore, we have thoroughly screened the entire Results section and removed or rephrased other similar methodological statements (such as details regarding primer design, specific staining protocols, and assay execution procedures) to ensure this section remains strictly focused on describing the biological findings.

4. The results section should not contain data interpretation. Authors are invited to describe experiments results without any interpretation. "This temporal dissociation suggests that the global UPR is likely a concurrent or secondary response rather than the primary early trigger of reproductive failure. Instead, our data suggest that early reproductive senescence is more likely driven by a breakdown in localized germline protein quality control, specifically through impaired lysosomal degradative capacity [6,19,41]."

"These data indicate that the W690C mutation imposes a substantial burden on lysosomal biogenesis and system-wide function."

We completely agree with the reviewer's rigorous standard that the Results section should remain strictly descriptive.

In the revised manuscript, we have removed the specific interpretive statements highlighted by the reviewer. Furthermore, we conducted a comprehensive review of the entire Results section and systematically removed all other instances of data interpretation, theoretical speculation, and broad conclusions, including statements regarding the validation of the model, extrapolations about organismal homeostasis, and conclusions about therapeutic potential.

All such interpretations and theoretical implications have now been carefully integrated into the Discussion section, where they are more appropriately contextualized. We believe this rigorous restructuring makes the presentation of our experimental data entirely objective, clean, and highly professional.

5.The discussion section is well written and appropriately explains the interest of the current study in establishing a comprehensive mechanistic framework for the link between LMD-3 mutation and premature ovarian insufficiency in C. elegans.

We sincerely thank the reviewer for their encouraging and positive assessment of our Discussion section. We are glad to hear that the contextualization of our findings provides a clear and comprehensive mechanistic framework for understanding the link between the LMD-3 mutation and premature ovarian insufficiency (POI).
