# Supplementary material for: A premature ovarian insufficiency-associated LMD-3 point mutation impairs protein stability and causes autophagic-lysosomal dysfunction in Caenorhabditis elegans
Source: PLoS One. 2026 Oct 27;21(10):e0361215. doi: 10.1371/journal.pone.0361215 (PMC13712518; doi:10.1371/journal.pone.0361215)
Supplement: Supplementary file 3 [file pone.0361215.s009.pdf]

Responses to Editorial Requests:

We sincerely thank the Academic Editor and the reviewer for their careful reading of our manuscript and for their valuable feedback. We have meticulously addressed each of the points raised in the editorial report, as detailed below:

1. *Please soften all statements referring to vitamin B12 "deficiency" and instead frame the findings as being "consistent with reduced vitamin B12 availability" or "consistent with impaired B12-dependent metabolism.*

We have thoroughly screened the manuscript to remove definitive or absolute references to systemic vitamin B12 "deficiency". All relevant statements across the **Abstract**, **Results**, and **Discussion** have been appropriately moderated and reframed as being "consistent with reduced vitamin B12 availability" or "consistent with impaired B12-dependent metabolism."

2. *Please avoid presenting AlphaFold3-based structural modeling as experimental evidence.*

We have carefully revised our text to ensure that the AlphaFold3-based structural predictions are strictly presented as computational models and hypothesis-generating analyses rather than direct experimental evidence of protein oligomerization or structural disruption.

3. *Please clarify that the cycloheximide-chase experiment performed in HEK293T cells supports an effect of the mutation on protein stability, but does not directly demonstrate altered endogenous LMD-3 stability in C. elegans.*

We have clarified throughout the text (including the **Results** and **Discussion**) that the cycloheximide (CHX)-chase assay was performed in heterologous HEK293T cells using exogenously expressed FLAG-tagged LMD-3, providing supportive evidence for intrinsic protein stability effects rather than direct measurement of endogenous LMD-3 stability in *C. elegans*.

4. *Please revise the interpretation of the qRT-PCR data to indicate "reduced steady-state transcript abundance," rather than implying transcriptional repression unless direct evidence is available.*

We have replaced absolute terms implying transcriptional repression (such as "suppresses gene expression") with more precise wording indicating **"reduced steady-state transcript abundance"** across the manuscript.

5. *Please clarify whether correction for multiple comparisons was performed. Alternatively, if the analyzed genes were predefined candidate targets, this should be explicitly stated in the Methods and/or figure legends.*

We have explicitly clarified in the **Materials and Methods** and **figure legends** that the analyzed lysosomal and autophagy-related genes were predefined candidate targets evaluated using targeted individual hypothesis tests.

6. *Please ensure that the Discussion is appropriately contextualized within the relevant recent literature on lysosomal dysfunction, autophagy, vitamin B12-related metabolism, and reproductive decline. Where directly relevant, the authors may consider incorporating additional recent studies, including 10.33594/000000744 and 10.1186/s43088-025-00604-2. These references should be discussed in relation to the manuscript's mechanistic interpretation, rather than added as isolated citations.*

We have significantly enriched the **Discussion** section by appropriately contextualizing our findings within the broader literature on lysosomal dysfunction, autophagy, and reproductive decline. Specifically, we have incorporated and discussed the suggested recent studies (DOI: 10.1186/s43088-025-00604-2 and DOI: 10.33594/000000744, as new References **[59]** and **[68]** respectively) in direct relation to our mechanistic interpretations. In addition, all other minor interpretative caveats raised by Reviewer #1, including distinguishing the mechanistic model from linear epistasis for vha-12, moderating explicit steps in autophagic flux, and separating experimentally demonstrated findings from proposed models, have been fully integrated and refined throughout the revised manuscript.

Response to Reviewer #1:
We sincerely thank the Reviewer for the constructive feedback and positive evaluation of our previous revisions. We have fully addressed the remaining points raised, focusing on refining mechanistic phrasing, moderating absolute claims, and enhancing interpretive precision. Our point-by-point responses are detailed below:

The authors have made substantial and constructive revisions in response to the previous review. The increased number of biological replicates, inclusion of effect-size measurements, improvement of figure quality, provision of raw Western blot data, and expanded discussion of the limitations of the C. elegans model have strengthened the manuscript considerably. I also appreciate the authors' efforts to moderate several of the translational and therapeutic statements. Nevertheless, a few points should be addressed before publication:
1. Vitamin B12 interpretation: *The acdh-1p::GFP reporter provides evidence consistent with altered vitamin B12 availability, but it does not directly demonstrate systemic vitamin B12 deficiency. Although this limitation is now acknowledged in the Discussion, some statements in the Results and Conclusion remain stronger than the available evidence. Please consistently frame the B12 phenotype as being "consistent with reduced vitamin B12 availability or impaired B12-dependent metabolism", rather than as definitively demonstrating systemic B12 deficiency.*

We completely agree with the reviewer's insightful critique. While the robust induction of the *acdh-1*p::GFP biosensor indicates an impairment in B12-

dependent metabolism, we acknowledge that it does not directly prove absolute systemic deficiency.

To ensure consistency and scientific rigor across the entire manuscript, we have conducted a thorough, systematic screening of the **Results, Discussion, and Conclusion** sections. All definitive or absolute references to systemic vitamin B12 "deficiency" have been removed or moderated and reframed as being "consistent with reduced vitamin B12 availability" or "consistent with impaired B12-dependent metabolism." We believe these modifications accurately represent our experimental findings while strictly adhering to the limits of our *in vivo* biosensor data.

2. Causal relationship between lysosomal dysfunction and B12 deficiency: *The mecobalamin rescue experiments are interesting and support a functional relationship between B12 metabolism and the observed phenotypes. However, they do not establish a definitive linear pathway in which lysosomal dysfunction causes B12 deficiency, which then causes autophagic and reproductive defects. Please distinguish clearly between experimentally demonstrated findings and the proposed mechanistic model.*

We appreciate this insightful distinction. We agree that the mecobalamin rescue experiments demonstrate a clear functional link rather than a rigid linear pathway. To address this, we have revised the **Discussion** to clearly separate our solid experimental findings, such as lysosomal failure, B12 biosensor activation, and meCbl-mediated rescue, from our working mechanistic model. We now frame altered B12 metabolism as a significant contributing factor operating alongside primary proteostatic collapse, rather than as a strict, single-track linear chain of events.

3. Structural modeling: *The AlphaFold3-based structural analyses and predicted trimer-like assembly are useful for generating mechanistic hypotheses. However, computational predictions should not be presented as experimental evidence of oligomerization, structural disruption, or a direct mechanism of protein degradation. Please moderate the relevant statements accordingly.*

We completely agree with the reviewer's prudent distinction. While computational modeling provides valuable structural insights for hypothesis generation, it cannot replace direct experimental proof.

To ensure absolute scientific rigor, we have carefully reviewed and refined all relevant text across the manuscript. We have explicitly ensured that the AlphaFold3-based structural analyses, predicted trimer assemblies, and interface distance measurements are strictly framed as computational predictions and hypothesis-generating models rather than direct experimental evidence of oligomerization, structural disruption, or protein degradation mechanisms. We believe these modifications appropriately calibrate the interpretation of our structural modeling data.

4. Protein stability experiment: *The cycloheximide-chase experiment was performed in HEK293T cells using exogenously expressed FLAG-tagged LMD-3. This is supportive evidence for an intrinsic effect of the W690C substitution on protein stability, but it does not directly demonstrate altered endogenous LMD-3 stability in C. elegans. Please maintain this distinction throughout the manuscript.*

We completely agree with this important distinction. While the cycloheximide (CHX)-chase assay in heterologous HEK293T cells provides valuable supportive evidence regarding the intrinsic impact of the W690C mutation on protein stability, we acknowledge that it does not directly measure endogenous LMD-3 protein turnover in *C. elegans*.
To ensure absolute scientific rigor and precision, we have carefully reviewed the text across the manuscript, particularly in the **Results** and **Discussion** sections, to maintain this exact distinction. We have ensured that our description strictly frames these heterologous results as supportive biochemical evidence for intrinsic protein stability effects, rather than overstating them as direct in vivo measurements of endogenous protein degradation within nematodes.

5. Interpretation of qRT-PCR: *Reduced steady-state lmd-3 mRNA abundance does not by itself demonstrate transcriptional repression. Statements such as "the mutation suppresses gene expression" should therefore be replaced with more precise wording indicating reduced steady-state transcript abundance, unless additional transcriptional evidence is available.*

We completely agree with the reviewer's perceptive distinction. qRT-PCR measures steady-state mRNA abundance, which reflects a net balance between transcription and degradation rates rather than strictly proving direct transcriptional repression.
To ensure absolute scientific rigor and precision, we have systematically screened the entire manuscript. All terms implying direct transcriptional repression or suppression, such as "suppresses gene expression" or "transcriptional downregulation", have been replaced with precise phrasing indicating "reduced steady-state transcript abundance" or "decreased steady-state mRNA levels".

6. Autophagy interpretation: *The tandem mCherry::GFP::LGG-1 reporter and LGG-1 measurements support impaired autophagic maturation and/or degradation. However, the precise molecular step at which the defect occurs is not completely established. Please avoid overly definitive statements regarding a block specifically at autophagosome–lysosome fusion or degradation.*

We completely agree with the reviewer's suggestion. While our tandem mCherry::GFP::LGG-1 reporter data and LGG-1 measurements clearly show impaired autophagic maturation and reduced degradation, we acknowledge that confirming the exact molecular step between autophagosome-lysosome fusion and subsequent degradation requires further study.

To ensure scientific rigor, we have carefully reviewed the manuscript and softened all overly definitive statements regarding the precise locus of the autophagic block. We now describe the defect more broadly as impaired autophagic maturation and degradation or compromised late-stage autophagic flux, rather than overstating a specific block at the fusion or degradation stage.

7. Genetic interaction with vha-12: *The vha-12 genetic experiment supports a functional relationship between LMD-3 and lysosomal V-ATPase-dependent function, but it does not establish direct molecular regulation or definitive linear epistasis. The interpretation should remain consistent with the authors' revised description of this relationship as a working mechanistic model.*

We completely agree with the reviewer's suggestion. While our *vha-12* genetic data demonstrate a clear functional relationship between LMD-3 and V-ATPase, we acknowledge that these experiments do not establish direct molecular regulation or definitive linear epistasis.
To ensure scientific rigor, we have carefully reviewed the manuscript to ensure that this relationship is consistently framed as a working mechanistic model rather than overstating direct molecular regulation or strict epistasis.

8. Statistical analysis: *For the multiple lysosomal and autophagy-related genes analyzed individually, please clarify whether correction for multiple comparisons was performed. If these genes were predefined candidate targets and individual hypothesis tests were intentionally used, this should be explicitly stated in the Methods and/or figure legends. Subject to appropriate clarification and moderation of these points, I consider the revised manuscript substantially improved. The remaining issues are primarily related to the precision of mechanistic interpretation rather than fundamental experimental deficiencies.*

We thank the reviewer for this constructive comment. These genes were predefined candidate targets, so we intentionally used individual hypothesis tests without correction for multiple comparisons.
We have added this statement to **the Statistical analysis section of the Methods**:
"For targeted transcript analyses (such as lysosomal and autophagy-related genes in Figs 4A and 5B), the evaluated genes were treated as predefined candidate targets and assessed using targeted individual hypothesis tests without multiple testing corrections."
We appreciate the reviewer's positive feedback.
