# Supplementary material for: A premature ovarian insufficiency-associated LMD-3 point mutation impairs protein stability and causes autophagic-lysosomal dysfunction in Caenorhabditis elegans
Source: PLoS One. 2026 Oct 27;21(10):e0361215. doi: 10.1371/journal.pone.0361215 (PMC13712518; doi:10.1371/journal.pone.0361215)
Supplement: Supplementary file 4 [file pone.0361215.s006.docx]

Fig 3B

180

IB: mCherry

130

100

55

70

40

35

25

IB: mCherry

100

70

55

40

Uncropped Western blot images for Fig 3B. mCherry(LMD-3) and mCherry(TLDc) were run on parallel gels.

35

25

Fig 5F

wt W690C

70

IB: GFP

55

40

35

wt W690C

70

55

IB: tubulin

40

Uncropped Western blot images for Fig 5F. GFP and tubulin were run on parallel gels.

S4F Fig

wt W690C

70

55

40

IB: GFP

35

wt W690C

70

55

40

IB: tubulin

Uncropped Western blot images for S4F Fig. GFP and tubulin were run on parallel gels.

Fig 7E

wild type	LMD^W690C^

130

IB: FLAG

IB: FLAG

130

100

100

70

70

GAPDH	GAPDH

IB: GAPDH

40

35

IB: GAPDH

40

35

Uncropped Western blot images for Fig 7E. FLAG and GAPDH were run on parallel gels.

S6B Fig

wild type	LMD^W690C^

130

IB: FLAG

130

100

70

100

70

IB: FLAG

GAPDH	GAPDH

40

35

IB: GAPDH

40

35

IB: GAPDH

Uncropped Western blot images for S6B Fig. FLAG and GAPDH were run on parallel gels.
