# Supplementary material for: A premature ovarian insufficiency-associated LMD-3 point mutation impairs protein stability and causes autophagic-lysosomal dysfunction in Caenorhabditis elegans
Source: PLoS One. 2026 Oct 27;21(10):e0361215. doi: 10.1371/journal.pone.0361215 (PMC13712518; doi:10.1371/journal.pone.0361215)
Supplement: Peer review history

# Peer review: Decision Letter 0

**DOI:** 10.1371/journal.pone.0361215.r001

**Authors:** Ahmed Atwa

29 Jul 2026

Dear Dr. Zhang,

Thank you for submitting your manuscript to PLOS ONE. After careful consideration, we feel that it has merit but does not fully meet PLOS ONE’s publication criteria as it currently stands. Therefore, we invite you to submit a revised version of the manuscript that addresses the points raised during the review process.

==============================

Dear Authors,

Thank you for submitting your manuscript entitled “A premature ovarian insufficiency-associated LMD-3 point mutation impairs protein stability and causes autophagic-lysosomal dysfunction in *Caenorhabditis elegans* ” to PLOS ONE.

The reviewers found the study interesting and biologically relevant, and they acknowledged the value of establishing a *C. elegans*  model carrying a POI-associated LMD-3/NCOA7 point mutation. The manuscript provides potentially important insights into the relationship between LMD-3 protein stability, lysosomal/autophagic dysfunction, proteostasis, germline apoptosis, and reproductive decline.

However, both reviewers identified several issues that must be addressed before the manuscript can be considered further. In particular, the mechanistic and translational conclusions require moderation and/or stronger support. The claims linking LMD-3 mutation, V-ATPase dysfunction, autophagy impairment, functional vitamin B12 deficiency, and reproductive decline should be presented more cautiously unless additional direct or genetic evidence is provided. The reviewers also requested clarification of the reduced LMD-3 transcript level, stronger support for the proposed vitamin B12-related mechanism, improved distinction between Results and Discussion, and greater emphasis on the limitations of extrapolating findings from *C. elegans*  to human POI.

Therefore, I invite you to submit a substantially revised manuscript. Please provide a detailed point-by-point response to all reviewer comments and revise the manuscript accordingly. In particular, please ensure that:

1. The manuscript should moderate its translational claims: the current *C. elegans*  data support a disease-relevant model, but they do not yet establish vitamin B12/mecobalamin as a directly translatable therapeutic intervention for human POI.
2. The proposed LMD-3–V-ATPase–lysosome–autophagy–reproductive decline pathway is interesting, but the causal hierarchy remains incompletely demonstrated; the authors should either add genetic/functional validation or clearly frame this as a proposed mechanistic model.
3. The reported reduction in both LMD-3 protein stability and transcript expression requires clarification, as the mechanism underlying decreased mRNA levels is not addressed and may affect interpretation of the mutation as primarily protein-destabilizing.
4. The vitamin B12 deficiency claim should be softened unless supported by direct biochemical measurements of B12 status or related metabolic markers; current evidence appears functional and indirect.
5. The Results section should be revised to remove methodological explanations and mechanistic interpretation, reserving such interpretation for the Discussion, with clearer separation between observed data and proposed biological mechanisms.

==============================

Please submit your revised manuscript by Sep 27 2026 11:59PM. If you will need more time than this to complete your revisions, please reply to this message or contact the journal office at plosone@plos.org. When you're ready to submit your revision, log on to https://www.editorialmanager.com/pone/ and select the 'Submissions Needing Revision' folder to locate your manuscript file.

- A letter that responds to each point raised by the academic editor and reviewer(s). You should upload this letter as a separate file labeled 'Response to Reviewers'.
- A marked-up copy of your manuscript that highlights changes made to the original version. You should upload this as a separate file labeled 'Revised Manuscript with Track Changes'.
- An unmarked version of your revised paper without tracked changes. You should upload this as a separate file labeled 'Manuscript'.

If you would like to make changes to your financial disclosure, please include your updated statement in your cover letter. Guidelines for resubmitting your figure files are available below the reviewer comments at the end of this letter.

If applicable, we recommend that you deposit your laboratory protocols in protocols.io to enhance the reproducibility of your results. Protocols.io assigns your protocol its own identifier (DOI) so that it can be cited independently in the future. For instructions see: https://journals.plos.org/plosone/s/submission-guidelines#loc-laboratory-protocols. Additionally, PLOS ONE offers an option for publishing peer-reviewed Lab Protocol articles, which describe protocols hosted on protocols.io. Read more information on sharing protocols at https://plos.org/protocols?utm_medium=editorial-email&utm_source=authorletters&utm_campaign=protocols.

As the corresponding author, your ORCID iD is verified in the submission system and will appear in the published article. PLOS supports the use of ORCID, and we encourage all coauthors to register for an ORCID iD and use it as well. Please encourage your coauthors to verify their ORCID iD within the submission system before final acceptance, as unverified ORCID iDs will not appear in the published article. *Only*  the individual author can complete the verification step; PLOS staff *cannot*  verify ORCID iDs on behalf of authors.

We look forward to receiving your revised manuscript.

Kind regards,

Ahmed Atwa, Ph.D

Academic Editor

PLOS One

Journal Requirements:

When submitting your revision, we need you to address these additional requirements.

1. Please ensure that your manuscript meets PLOS ONE's style requirements, including those for file naming. The PLOS ONE style templates can be found at

https://journals.plos.org/plosone/s/file?id=wjVg/PLOSOne_formatting_sample_main_body.pdf and https://journals.plos.org/plosone/s/file?id=ba62/PLOSOne_formatting_sample_title_authors_affiliations.pdf

2. PLOS ONE now requires that authors provide the original uncropped and unadjusted images underlying all blot or gel results reported in a submission’s figures or Supporting Information files. This policy and the journal’s other requirements for blot/gel reporting and figure preparation are described in detail at https://journals.plos.org/plosone/s/figures#loc-blot-and-gel-reporting-requirements and https://journals.plos.org/plosone/s/figures#loc-preparing-figures-from-image-files. When you submit your revised manuscript, please ensure that your figures adhere fully to these guidelines and provide the original underlying images for all blot or gel data reported in your submission. See the following link for instructions on providing the original image data: https://journals.plos.org/plosone/s/figures#loc-original-images-for-blots-and-gels.

In your cover letter, please note whether your blot/gel image data are in Supporting Information or posted at a public data repository, provide the repository URL if relevant, and provide specific details as to which raw blot/gel images, if any, are not available. Email us at plosone@plos.org if you have any questions.

3. We are unable to open your Supporting Information file [Supporting information for lmd-3(W690C)_PLoS one word.rar]. Please kindly revise as necessary and re-upload.

4. If the reviewer comments include a recommendation to cite specific previously published works, please review and evaluate these publications to determine whether they are relevant and should be cited. There is no requirement to cite these works unless the editor has indicated otherwise.

[Note: HTML markup is below. Please do not edit.]

Reviewers' comments:

Reviewer's Responses to Questions

**Comments to the Author**

1. Is the manuscript technically sound, and do the data support the conclusions?

Reviewer #1: Partly

Reviewer #2: Yes

**********

2. Has the statistical analysis been performed appropriately and rigorously?

Reviewer #1: N/A

Reviewer #2: Yes

**********

3. Have the authors made all data underlying the findings in their manuscript fully available?

The PLOS Data policy

Reviewer #1: Yes

Reviewer #2: Yes

**********

4. Is the manuscript presented in an intelligible fashion and written in standard English?

Reviewer #1: No

Reviewer #2: Yes

**********

Reviewer #1: Recommendation: Major Revision

Major Comments

1. Although the study successfully establishes a C. elegans model carrying the POI-associated mutation, the translational relevance to human disease remains insufficiently supported. The manuscript would benefit from additional experimental evidence or a more cautious interpretation of the findings regarding human POI.

2. The authors report that the W690C mutation reduces both LMD-3 protein stability and transcript levels. However, the mechanism underlying the reduction in mRNA expression is not investigated. Additional experiments or a more detailed discussion addressing this observation would strengthen the mechanistic conclusions.

3. The conclusion that lysosomal dysfunction induces a functional vitamin B12 deficiency is based primarily on indirect evidence. Direct biochemical measurements of vitamin B12 levels or related metabolic markers would provide stronger support for this important claim.

4. The proposed mechanistic pathway linking LMD-3, V-ATPase dysfunction, autophagy impairment, and reproductive decline would be strengthened by additional genetic interaction (epistasis) experiments or complementary functional analyses.

5. Several conclusions, particularly those suggesting that vitamin B12 represents a "translatable therapeutic intervention," appear stronger than the presented data support. Since all experiments were performed in C. elegans, these statements should be moderated.

Minor Comments

1. The quality of several fluorescence microscopy images should be improved to facilitate data interpretation.

2. Some quantitative analyses, particularly RT-qPCR experiments with only three biological replicates, would benefit from additional replication to improve statistical robustness.

3. The Discussion should further emphasize the limitations of the C. elegans model when extrapolating findings to human ovarian biology.

4. Several sentences in the Introduction and Discussion are overly long and could be revised for improved readability.

5. Reporting effect sizes in addition to p-values would improve the statistical presentation of the results.

Overall Evaluation

This manuscript addresses an interesting and biologically relevant question using a comprehensive experimental approach. The data are generally of good quality, and the findings provide valuable insights into the role of LMD-3 in lysosomal function and reproductive aging. However, several mechanistic claims require stronger experimental support, and some conclusions should be presented more cautiously. Therefore, I recommend Major Revision before the manuscript can be considered for publication.

Reviewer #2: This manuscript addresses a relevant topic—the impact of LMD-3 point mutation on premature ovarian insufficiency in C. elegans.

The introduction is well structured and allows a comprehensive overview of the recent advances related to the topic. However, authors described the findings of the current study at the end of the introduction section. These data are not relevant to the introduction, they should be removed “Our data show that this point mutation significantly reduces early reproductive capacity…..a potential therapeutic intervention”.

Authors should clearly formulate the aim of the study at the end of the introduction section.

Some methodological points are stated in the results section (example: Employing two complementary approaches—the nucleic acid dye SYTO12 and a lim-7p::ced-1::GFP reporter [35,36], we observed….These data are to remove as they are related to the methods section.

The sesults section should not contain data interpretation. Authors are invited to describe experiments results without anay interpretation. “This temporal dissociation suggests that the global UPR is likely a concurrent or secondary response rather than the primary early trigger of reproductive failure. Instead, our data sugges that early reproductive senescence is more likely driven by a breakdown in localized germline protein quality control, specifically through impaired lysosomal degradative capacity [6,19,41].”

“These data indicate that the W690C mutation imposes a substantial burden on lysosomal biogenesis and system-wide function. “

The discussion section is well written and appropriatly explains the interest of the current study in establishing a comprehensive mechanistic framework for the link between LMD-3 mutation and premature ovarian insufficiency in C. elegans.

**********

what does this mean?). If published, this will include your full peer review and any attached files.

If you choose “no”, your identity will remain anonymous but your review may still be made public.

**Do you want your identity to be public for this peer review?**  For information about this choice, including consent withdrawal, please see our Privacy Policy

Reviewer #1: **Yes:**  Ali Salehi

Reviewer #2: No

**********

[NOTE: If reviewer comments were submitted as an attachment file, they will be attached to this email and accessible via the submission site. Please log into your account, locate the manuscript record, and check for the action link "View Attachments". If this link does not appear, there are no attachment files.]

To ensure your figures meet our technical requirements, please review our figure guidelines: https://journals.plos.org/plosone/s/figures

You may also use PLOS’s free figure tool, NAAS, to help you prepare publication quality figures: https://journals.plos.org/plosone/s/figures#loc-tools-for-figure-preparation.

NAAS will assess whether your figures meet our technical requirements by comparing each figure against our figure specifications.

# Author comment: Author response to Decision Letter 1

**DOI:** 10.1371/journal.pone.0361215.r002

13 Aug 2026

Response to Reviews:

We are grateful to the reviewers for their constructive and insightful feedback on our manuscript. We are encouraged that they recognized the value of our C. elegans POI model. In response to their suggestions, we have increased RT-qPCR biological replicates, optimized image quality, and included effect size measurements. More importantly, we have restructured the Results and Discussion sections, systematically moderated our translational and vitamin B12-related claims, framed the proposed pathway as a working mechanistic model, and resolved all technical formatting and raw data requirements.

A detailed, point-by-point response to all comments, along with the marked-up and clean manuscript files, has been fully uploaded as an attachment ('Response to Reviewers'). For quick reference, a summary of our point-by-point responses is also provided below:

Reviewer #1: Recommendation: Major Revision

Major Comments:

Regarding the translational relevance to human disease, we have systematically softened our claims on vitamin B12 as a direct clinical intervention, framing it instead as a conceptual metabolic lead, and expanded the Discussion to explicitly outline the biological and physiological boundaries of extrapolating C. elegans data to human ovarian biology.

Regarding the reduced lmd-3 mRNA expression, we increased our RT-qPCR biological replicates from n=3 to n=4 (now in updated Fig 7B), added a discussion on theoretical transcript surveillance mechanisms, and addressed species-specific variations.

Regarding the indirect evidence of vitamin B12 deficiency, we softened the language in the Results to "strongly indicating" and explicitly acknowledged the reliance on the acdh-1 biosensor as a limitation in the Discussion.

Regarding the genetic interaction pathway, we have contextualized the proposed cascade as a working mechanistic model rather than proven linear epistasis, keeping the focus on variant-specific biochemical collapse and metabolic rescue.

Regarding the moderation of therapeutic claims, we have thoroughly tempered our conclusions in the Abstract and Discussion to avoid overstating clinical efficacy.

Minor Comments:

We have re-evaluated and optimized the resolution, contrast, and clarity of key fluorescence images (Fig 4H-I, Fig 5C, S3F Fig).

We have increased RT-qPCR biological replicates from n=3 to n=4 across all transcriptional analyses (Fig 4A, 5B, 7B).

We have expanded the Discussion to thoroughly emphasize the limitations of the C. elegans model.

We have systematically shortened and clarified overly long sentences in the Introduction and Discussion.

We have calculated and reported effect sizes (Cohen's d) for quantitative comparisons in the figure legends and S1 Data.

Reviewer #2:

1 & 2. We have removed the summary of findings from the Introduction and replaced it with a clear formulation of the study's aims.

3. We have removed methodological statements from the Results section.

4. We have removed data interpretations and speculations from the Results section to ensure purely objective data reporting.

5. We sincerely thank the reviewer for the encouraging assessment of our Discussion section.

Attachment Submitted filename: 4. Response to Reviews_PLoS one_RV1.pdf

# Peer review: Decision Letter 1

**DOI:** 10.1371/journal.pone.0361215.r003

**Authors:** Ahmed Atwa

5 Oct 2026

Dear Dr. Zhang,

Thank you for submitting your manuscript to PLOS One. After careful consideration, we feel that it has merit but does not fully meet PLOS One’s publication criteria as it currently stands. Therefore, we invite you to submit a revised version of the manuscript that addresses the points raised during the review process.

==============================

**The main points that should be addressed are as follows:**

1. Please soften all statements referring to vitamin B12 “deficiency” and instead frame the findings as being “consistent with reduced vitamin B12 availability” or “consistent with impaired B12-dependent metabolism.”
2. Please avoid presenting AlphaFold3-based structural modeling as experimental evidence.
3. Please clarify that the cycloheximide-chase experiment performed in HEK293T cells supports an effect of the mutation on protein stability, but does not directly demonstrate altered endogenous LMD-3 stability in C. elegans.
4. Please revise the interpretation of the qRT-PCR data to indicate “reduced steady-state transcript abundance,” rather than implying transcriptional repression unless direct evidence is available.
5. Please clarify whether correction for multiple comparisons was performed. Alternatively, if the analyzed genes were predefined candidate targets, this should be explicitly stated in the Methods and/or figure legends.
6. Please ensure that the Discussion is appropriately contextualized within the relevant recent literature on lysosomal dysfunction, autophagy, vitamin B12-related metabolism, and reproductive decline. Where directly relevant, the authors may consider incorporating additional recent studies, including 10.33594/000000744 and 10.1186/s43088-025-00604-2. These references should be discussed in relation to the manuscript’s mechanistic interpretation, rather than added as isolated citations.

Overall, the appropriate decision is Minor Revision, with editorial assessment after revision. The manuscript appears close to acceptance, but the remaining mechanistic and interpretative statements should be refined before final acceptance.

==============================

Please submit your revised manuscript by Nov 04 2026 11:59PM. If you will need more time than this to complete your revisions, please reply to this message or contact the journal office at plosone@plos.org. When you're ready to submit your revision, log on to https://www.editorialmanager.com/pone/ and select the 'Submissions Needing Revision' folder to locate your manuscript file.

- A letter that responds to each point raised by the academic editor and reviewer(s). You should upload this letter as a separate file labeled 'Response to Reviewers'.
- A marked-up copy of your manuscript that highlights changes made to the original version. You should upload this as a separate file labeled 'Revised Manuscript with Track Changes'.
- An unmarked version of your revised paper without tracked changes. You should upload this as a separate file labeled 'Manuscript'.

If you would like to make changes to your financial disclosure, please include your updated statement in your cover letter. Guidelines for resubmitting your figure files are available below the reviewer comments at the end of this letter.

If applicable, we recommend that you deposit your laboratory protocols in protocols.io to enhance the reproducibility of your results. Protocols.io assigns your protocol its own identifier (DOI) so that it can be cited independently in the future. For instructions see: https://journals.plos.org/plosone/s/submission-guidelines#loc-laboratory-protocols. Additionally, PLOS One offers an option for publishing peer-reviewed Lab Protocol articles, which describe protocols hosted on protocols.io. Read more information on sharing protocols at https://plos.org/protocols?utm_medium=editorial-email&utm_source=authorletters&utm_campaign=protocols.

As the corresponding author, your ORCID iD is verified in the submission system and will appear in the published article. PLOS supports the use of ORCID, and we encourage all coauthors to register for an ORCID iD and use it as well. Please encourage your coauthors to verify their ORCID iD within the submission system before final acceptance, as unverified ORCID iDs will not appear in the published article. *Only*  the individual author can complete the verification step; PLOS staff *cannot*  verify ORCID iDs on behalf of authors.

We look forward to receiving your revised manuscript.

Kind regards,

Ahmed Atwa, Ph.D

Academic Editor

PLOS One

**Journal Requirements:**

1. If the reviewer comments include a recommendation to cite specific previously published works, please review and evaluate these publications to determine whether they are relevant and should be cited. There is no requirement to cite these works unless the editor has indicated otherwise.

2. Please review your reference list to ensure that it is complete and correct. If you have cited papers that have been retracted, please include the rationale for doing so in the manuscript text, or remove these references and replace them with relevant current references. Any changes to the reference list should be mentioned in the rebuttal letter that accompanies your revised manuscript. If you need to cite a retracted article, indicate the article’s retracted status in the References list and also include a citation and full reference for the retraction notice.

[Note: HTML markup is below. Please do not edit.]

Reviewers' comments:

Reviewer's Responses to Questions

**Comments to the Author**

Reviewer #1: All comments have been addressed

**********

2. Is the manuscript technically sound, and do the data support the conclusions?

Reviewer #1: Partly

**********

3. Has the statistical analysis been performed appropriately and rigorously?

Reviewer #1: No

**********

4. Have the authors made all data underlying the findings in their manuscript fully available?

The PLOS Data policy

Reviewer #1: Yes

**********

5. Is the manuscript presented in an intelligible fashion and written in standard English?

Reviewer #1: Yes

**********

Reviewer #1: The authors have made substantial and constructive revisions in response to the previous review. The increased number of biological replicates, inclusion of effect-size measurements, improvement of figure quality, provision of raw Western blot data, and expanded discussion of the limitations of the C. elegans model have strengthened the manuscript considerably. I also appreciate the authors’ efforts to moderate several of the translational and therapeutic statements.

Nevertheless, a few points should be addressed before publication:

1. **Vitamin B12 interpretation:** The acdh-1p::GFP reporter provides evidence consistent with altered vitamin B12 availability, but it does not directly demonstrate systemic vitamin B12 deficiency. Although this limitation is now acknowledged in the Discussion, some statements in the Results and Conclusion remain stronger than the available evidence. Please consistently frame the B12 phenotype as being *consistent with reduced vitamin B12 availability or impaired B12-dependent metabolism*, rather than as definitively demonstrating systemic B12 deficiency.

2. **Causal relationship between lysosomal dysfunction and B12 deficiency:** The mecobalamin rescue experiments are interesting and support a functional relationship between B12 metabolism and the observed phenotypes. However, they do not establish a definitive linear pathway in which lysosomal dysfunction causes B12 deficiency, which then causes autophagic and reproductive defects. Please distinguish clearly between experimentally demonstrated findings and the proposed mechanistic model.

3. **Structural modeling:** The AlphaFold3-based structural analyses and predicted trimer-like assembly are useful for generating mechanistic hypotheses. However, computational predictions should not be presented as experimental evidence of oligomerization, structural disruption, or a direct mechanism of protein degradation. Please moderate the relevant statements accordingly.

4. **Protein stability experiment:** The cycloheximide-chase experiment was performed in HEK293T cells using exogenously expressed FLAG-tagged LMD-3. This is supportive evidence for an intrinsic effect of the W690C substitution on protein stability, but it does not directly demonstrate altered endogenous LMD-3 stability in C. elegans. Please maintain this distinction throughout the manuscript.

5. **Interpretation of qRT-PCR:** Reduced steady-state lmd-3 mRNA abundance does not by itself demonstrate transcriptional repression. Statements such as “the mutation suppresses gene expression” should therefore be replaced with more precise wording indicating reduced steady-state transcript abundance, unless additional transcriptional evidence is available.

6. **Autophagy interpretation:** The tandem mCherry::GFP::LGG-1 reporter and LGG-1 measurements support impaired autophagic maturation and/or degradation. However, the precise molecular step at which the defect occurs is not completely established. Please avoid overly definitive statements regarding a block specifically at autophagosome–lysosome fusion or degradation.

7. **Genetic interaction with vha-12:** The vha-12 genetic experiment supports a functional relationship between LMD-3 and lysosomal V-ATPase-dependent function, but it does not establish direct molecular regulation or definitive linear epistasis. The interpretation should remain consistent with the authors’ revised description of this relationship as a working mechanistic model.

8. **Statistical analysis:** For the multiple lysosomal and autophagy-related genes analyzed individually, please clarify whether correction for multiple comparisons was performed. If these genes were predefined candidate targets and individual hypothesis tests were intentionally used, this should be explicitly stated in the Methods and/or figure legends.

Subject to appropriate clarification and moderation of these points, I consider the revised manuscript substantially improved. The remaining issues are primarily related to the precision of mechanistic interpretation rather than fundamental experimental deficiencies.

**********

what does this mean?). If published, this will include your full peer review and any attached files.

If you choose “no”, your identity will remain anonymous but your review may still be made public.

**Do you want your identity to be public for this peer review?**  For information about this choice, including consent withdrawal, please see our Privacy Policy

Reviewer #1: No

**********

[NOTE: If reviewer comments were submitted as an attachment file, they will be attached to this email and accessible via the submission site. Please log into your account, locate the manuscript record, and check for the action link "View Attachments". If this link does not appear, there are no attachment files.]

To ensure your figures meet our technical requirements, please review our figure guidelines: https://journals.plos.org/plosone/s/figures

You may also use PLOS’s free figure tool, NAAS, to help you prepare publication quality figures: https://journals.plos.org/plosone/s/figures#loc-tools-for-figure-preparation.

NAAS will assess whether your figures meet our technical requirements by comparing each figure against our figure specifications.

# Author comment: Author response to Decision Letter 2

**DOI:** 10.1371/journal.pone.0361215.r004

6 Oct 2026

Responses to Editorial Requests:

We sincerely thank the Academic Editor and the reviewer for their careful reading of our manuscript and for their valuable feedback. We have meticulously addressed each of the points raised in the editorial report, as detailed below:

1. Please soften all statements referring to vitamin B12 “deficiency” and instead frame the findings as being “consistent with reduced vitamin B12 availability” or “consistent with impaired B12-dependent metabolism.

We have thoroughly screened the manuscript to remove definitive or absolute references to systemic vitamin B12 "deficiency". All relevant statements across the Abstract, Results, and Discussion have been appropriately moderated and reframed as being "consistent with reduced vitamin B12 availability" or "consistent with impaired B12-dependent metabolism."

2. Please avoid presenting AlphaFold3-based structural modeling as experimental evidence.

We have carefully revised our text to ensure that the AlphaFold3-based structural predictions are strictly presented as computational models and hypothesis-generating analyses rather than direct experimental evidence of protein oligomerization or structural disruption.

3. Please clarify that the cycloheximide-chase experiment performed in HEK293T cells supports an effect of the mutation on protein stability, but does not directly demonstrate altered endogenous LMD-3 stability in C. elegans.

We have clarified throughout the text (including the Results and Discussion) that the cycloheximide (CHX)-chase assay was performed in heterologous HEK293T cells using exogenously expressed FLAG-tagged LMD-3, providing supportive evidence for intrinsic protein stability effects rather than direct measurement of endogenous LMD-3 stability in C. elegans.

4. Please revise the interpretation of the qRT-PCR data to indicate “reduced steady-state transcript abundance,” rather than implying transcriptional repression unless direct evidence is available.

We have replaced absolute terms implying transcriptional repression (such as "suppresses gene expression") with more precise wording indicating "reduced steady-state transcript abundance" across the manuscript.

5. Please clarify whether correction for multiple comparisons was performed. Alternatively, if the analyzed genes were predefined candidate targets, this should be explicitly stated in the Methods and/or figure legends.

We have explicitly clarified in the Materials and Methods and figure legends that the analyzed lysosomal and autophagy-related genes were predefined candidate targets evaluated using targeted individual hypothesis tests.

6. Please ensure that the Discussion is appropriately contextualized within the relevant recent literature on lysosomal dysfunction, autophagy, vitamin B12-related metabolism, and reproductive decline. Where directly relevant, the authors may consider incorporating additional recent studies, including 10.33594/000000744 and 10.1186/s43088-025-00604-2. These references should be discussed in relation to the manuscript’s mechanistic interpretation, rather than added as isolated citations.

We have significantly enriched the Discussion section by appropriately contextualizing our findings within the broader literature on lysosomal dysfunction, autophagy, and reproductive decline. Specifically, we have incorporated and discussed the suggested recent studies (DOI: 10.1186/s43088-025-00604-2 and DOI: 10.33594/000000744, as new References [59] and [68] respectively) in direct relation to our mechanistic interpretations. In addition, all other minor interpretative caveats raised by Reviewer #1, including distinguishing the mechanistic model from linear epistasis for vha-12, moderating explicit steps in autophagic flux, and separating experimentally demonstrated findings from proposed models, have been fully integrated and refined throughout the revised manuscript.

Response to Reviewer #1:

We sincerely thank the Reviewer for the constructive feedback and positive evaluation of our previous revisions. We have fully addressed the remaining points raised, focusing on refining mechanistic phrasing, moderating absolute claims, and enhancing interpretive precision. Our point-by-point responses are detailed below:

The authors have made substantial and constructive revisions in response to the previous review. The increased number of biological replicates, inclusion of effect-size measurements, improvement of figure quality, provision of raw Western blot data, and expanded discussion of the limitations of the C. elegans model have strengthened the manuscript considerably. I also appreciate the authors’ efforts to moderate several of the translational and therapeutic statements. Nevertheless, a few points should be addressed before publication:

1. Vitamin B12 interpretation: The acdh-1p::GFP reporter provides evidence consistent with altered vitamin B12 availability, but it does not directly demonstrate systemic vitamin B12 deficiency. Although this limitation is now acknowledged in the Discussion, some statements in the Results and Conclusion remain stronger than the available evidence. Please consistently frame the B12 phenotype as being “consistent with reduced vitamin B12 availability or impaired B12-dependent metabolism”, rather than as definitively demonstrating systemic B12 deficiency.

We completely agree with the reviewer’s insightful critique. While the robust induction of the acdh-1p::GFP biosensor indicates an impairment in B12-dependent metabolism, we acknowledge that it does not directly prove absolute systemic deficiency.

To ensure consistency and scientific rigor across the entire manuscript, we have conducted a thorough, systematic screening of the Results, Discussion, and Conclusion sections. All definitive or absolute references to systemic vitamin B12 "deficiency" have been removed or moderated and reframed as being "consistent with reduced vitamin B12 availability" or "consistent with impaired B12-dependent metabolism." We believe these modifications accurately represent our experimental findings while strictly adhering to the limits of our in vivo biosensor data.

2. Causal relationship between lysosomal dysfunction and B12 deficiency: The mecobalamin rescue experiments are interesting and support a functional relationship between B12 metabolism and the observed phenotypes. However, they do not establish a definitive linear pathway in which lysosomal dysfunction causes B12 deficiency, which then causes autophagic and reproductive defects. Please distinguish clearly between experimentally demonstrated findings and the proposed mechanistic model.

We appreciate this insightful distinction. We agree that the mecobalamin rescue experiments demonstrate a clear functional link rather than a rigid linear pathway. To address this, we have revised the Discussion to clearly separate our solid experimental findings, such as lysosomal failure, B12 biosensor activation, and meCbl-mediated rescue, from our working mechanistic model. We now frame altered B12 metabolism as a significant contributing factor operating alongside primary proteostatic collapse, rather than as a strict, single-track linear chain of events.

3. Structural modeling: The AlphaFold3-based structural analyses and predicted trimer-like assembly are useful for generating mechanistic hypotheses. However, computational predictions should not be presented as experimental evidence of oligomerization, structural disruption, or a direct mechanism of protein degradation. Please moderate the relevant statements accordingly.

We completely agree with the reviewer’s prudent distinction. While computational modeling provides valuable structural insights for hypothesis generation, it cannot replace direct experimental proof.

To ensure absolute scientific rigor, we have carefully reviewed and refined all relevant text across the manuscript. We have explicitly ensured that the AlphaFold3-based structural analyses, predicted trimer assemblies, and interface distance measurements are strictly framed as computational predictions and hypothesis-generating models rather than direct experimental evidence of oligomerization, structural disruption, or protein degradation mechanisms. We believe these modifications appropriately calibrate the interpretation of our structural modeling data.

4. Protein stability experiment: The cycloheximide-chase experiment was performed in HEK293T cells using exogenously expressed FLAG-tagged LMD-3. This is supportive evidence for an intrinsic effect of the W690C substitution on protein stability, but it does not directly demonstrate altered endogenous LMD-3 stability in C. elegans. Please maintain this distinction throughout the manuscript.

We completely agree with this important distinction. While the cycloheximide (CHX)-chase assay in heterologous HEK293T cells provides valuable supportive evidence regarding the intrinsic impact of the W690C mutation on protein stability, we acknowledge that it does not directly measure endogenous LMD-3 protein turnover in C. elegans.

To ensure absolute scientific rigor and precision, we have carefully reviewed the text across the manuscript, particularly in the Results and Discussion sections, to maintain this exact distinction. We have ensured that our description strictly frames these heterologous results as supportive biochemical evidence for intrinsic protein stability effects, rather than overstating them as direct in vivo measurements of endogenous protein degradation within nematodes.

5. Interpretation of qRT-PCR: Reduced steady-state lmd-3 mRNA abundance does not by itself demonstrate transcriptional repression. Statements such as “the mutation suppresses gene expression” should therefore be replaced with more precise wording indicating reduced steady-state transcript abundance, unless additional transcriptional evidence is available.

We completely agree with the reviewer’s perceptive distinction. qRT-PCR measures steady-state mRNA abundance, which reflects a net balance between transcription and degradation rates rather than strictly proving direct transcriptional repression.

To ensure absolute scientific rigor and precision, we have systematically screened the entire manuscript. All terms implying direct transcriptional repression or suppression, such as "suppresses gene expression" or "transcriptional downregulation", have been replaced with precise phrasing indicating "reduced steady-state transcript abundance" or "decreased steady-state mRNA levels".

6. Autophagy interpretation: The tandem mCherry::GFP::LGG-1 reporter and LGG-1 measurements support impaired autophagic maturation and/or degradation. However, the precise molecular step at which the defect occurs is not completely established. Please avoid overly definitive statements regarding a block specifically at autophagosome–lysosome fusion or degradation.

We completely agree with the reviewer’s suggestion. While our tandem mCherry::GFP::LGG-1 reporter data and LGG-1 measurements clearly show impaired autophagic maturation and reduced degradation, we acknowledge that confirming the exact molecular step between autophagosome-lysosome fusion and subsequent degradation requires further study.

To ensure scientific rigor, we have carefully reviewed the manuscript and softened all overly definitive statements regarding the precise locus of the autophagic block. We now describe the defect more broadly as impaired autophagic maturation and degradation or compromised late-stage autophagic flux, rather than overstating a specific block at the fusion or degradation stage.

7. Genetic interaction with vha-12: The vha-12 genetic experiment supports a functional relationship between LMD-3 and lysosomal V-ATPase-dependent function, but it does not establish direct molecular regulation or definitive linear epistasis. The interpretation should remain consistent with the authors’ revised description of this relationship as a working mechanistic model.

We completely agree with the reviewer’s suggestion. While our vha-12 genetic data demonstrate a clear functional relationship between LMD-3 and V-ATPase, we acknowledge that these experiments do not establish direct molecular regulation or definitive linear epistasis.

To ensure scientific rigor, we have carefully reviewed the manuscript to ensure that this relationship is consistently framed as a working mechanistic model rather than overstating direct molecular regulation or strict epistasis.

8. Statistical analysis: For the multiple lysosomal and autophagy-related genes analyzed individually, please clarify whether correction for multiple comparisons was performed. If these genes were predefined candidate targets and individual hypothesis tests were intentionally used, this should be explicitly stated in the Methods and/or figure legends. Subject to appropriate clarification and moderation of these points, I consider the revised manuscript substantially improved. The remaining issues are primarily related to the precision of mechanistic interpretation rather than fundamental experimental deficiencies.

We thank the reviewer for this constructive comment. These genes were predefined candidate targets, so we intentionally used individual hypothesis tests without correction for multiple comparisons.

We have added this statement to the Statistical analysis section of the Methods:

"For targeted transcript analyses (such as lysosomal and autophagy-related genes in Figs 4A and 5B), the evaluated genes were treated as predefined candidate targets and assessed using targeted individual hypothesis tests without multiple testing corrections."

We appreciate the reviewer's positive feedback.

Attachment Submitted filename: 4. Response to Reviewers_RV2.pdf

# Editor report: Decision Letter 2

**DOI:** 10.1371/journal.pone.0361215.r005

**Authors:** Ahmed Atwa

8 Oct 2026

A premature ovarian insufficiency-associated LMD-3 point mutation impairs protein stability and causes autophagic-lysosomal dysfunction in Caenorhabditis elegans

PONE-D-26-28276R2

Dear Dr. Zhang,

We’re pleased to inform you that your manuscript has been judged scientifically suitable for publication and will be formally accepted for publication once it meets all outstanding technical requirements.

Within one week, you’ll receive an e-mail detailing the required amendments. When these have been addressed, you’ll receive a formal acceptance letter and your manuscript will be scheduled for publication.

An invoice will be generated when your article is formally accepted. Please note, if your institution has a publishing partnership with PLOS and your article meets the relevant criteria, all or part of your publication costs will be covered. Please make sure your user information is up-to-date by logging into Editorial Manager at Editorial Manager® and clicking the ‘Update My Information' link at the top of the page. For questions related to billing, please contact billing support.

If your institution or institutions have a press office, please notify them about your upcoming paper to help maximize its impact. If they’ll be preparing press materials, please inform our press team as soon as possible -- no later than 48 hours after receiving the formal acceptance. Your manuscript will remain under strict press embargo until 2 pm Eastern Time on the date of publication. For more information, please contact onepress@plos.org.

Kind regards,

Ahmed Atwa, Ph.D

Academic Editor

PLOS One

Additional Editor Comments (optional):

Reviewers' comments:

# Editor report: Acceptance letter

**DOI:** 10.1371/journal.pone.0361215.r006

**Authors:** Ahmed Atwa

PONE-D-26-28276R2

PLOS One

Dear Dr. Zhang,

I'm pleased to inform you that your manuscript has been deemed suitable for publication in PLOS One. Congratulations! Your manuscript is now being handed over to our production team.

At this stage, our production department will prepare your paper for publication. This includes ensuring the following:

* All references, tables, and figures are properly cited

* All relevant supporting information is included in the manuscript submission,

* There are no issues that prevent the paper from being properly typeset

You will receive further instructions from the production team, including instructions on how to review your proof when it is ready. Please keep in mind that we are working through a large volume of accepted articles, so please give us a few days to review your paper and let you know the next and final steps.

Lastly, if your institution or institutions have a press office, please let them know about your upcoming paper now to help maximize its impact. If they'll be preparing press materials, please inform our press team within the next 48 hours. Your manuscript will remain under strict press embargo until 2 pm Eastern Time on the date of publication. For more information, please contact onepress@plos.org.

You will receive an invoice from PLOS for your publication fee after your manuscript has reached the completed accept phase. If you receive an email requesting payment before acceptance or for any other service, this may be a phishing scheme. Learn how to identify phishing emails and protect your accounts at https://explore.plos.org/phishing.

If we can help with anything else, please email us at customercare@plos.org.

Thank you for submitting your work to PLOS One and supporting open access.

Kind regards,

PLOS One Editorial Office Staff

on behalf of

Dr. Ahmed Atwa

Academic Editor

PLOS One
